# Efficiency of chlorine and UV in the inactivation of *Cryptosporidium* and *Giardia* in wastewater

**DOI:** 10.1371/journal.pone.0216040

**Published:** 2019-05-13

**Authors:** Folasade Esther Adeyemo, Gulshan Singh, Poovendhree Reddy, Faizal Bux, Thor Axel Stenström

**Affiliations:** 1 SARChI Chair, Institute for Water and Wastewater Technology (IWWT), Durban University of Technology, Durban, South Africa; 2 Department of Community Health Studies, Faculty of Health Sciences, Durban University of Technology, Durban, South Africa; Universidade de Aveiro, PORTUGAL

## Abstract

Wastewater from different sources is contaminated by protozoan parasites including *Cryptosporidium* and *Giardia*. Many protozoan parasites are becoming resistant to chemical treatment. The challenge of finding alternatives is presented to researchers by exploring other methods of eliminating protozoan parasites from wastewater. The aim of this study was to assess the speciation and the viability of *Cryptosporidium* and *Giardia* in environmental samples with the specific objective of evaluating if effluent chlorination and UV affect the viability. Different doses of chlorine with different exposure times were experimented with both distilled water and waste water spiked with (oo)cysts derived from environmental samples. UV irradiation at different doses was also experimented using the same spiked samples. Two methods of quantification and detection, namely, microscopy and flow cytometry, were used in the experiment. Two vital dyes, Syto-9+PI and DAPI+PI, were the used for staining the collected wastewater samples. It was found that the (oo)cysts responded to chlorination and UV treatments with *Giardia* responding better than *Cryptosporidium*. Giardia responded very well to UV irradiations with almost 0 percent remaining viable after a low dose of UV. *Cryptosporidium* was found to be resistant to chlorination even at high doses but responded well to high UV doses. DAPI+PI dye gave a lower mean percentage viability values than Syto-9+PI. Flow cytometry gave higher mean percentage than microscopy from the results. It is concluded that UV is a promising alternative to Chlorine in removing *Cryptosporidium* and *Giardia* from waste water. Appropriate treatment method for wastewater is necessary to minimize water resources pollution when wastewater is released into water systems.

## Introduction

There is a great concern for the safety and security of environmental water which invariably affects the quality of drinking water and aquatic habitats. Water may be contaminated by environmental pollution and industrial activities. Environmental pollution may result from land use around water sources especially along informal settlements of developing countries. Some small-scale agricultural activities around water sources may also introduce contaminants in the runoff flowing into rivers, ponds and lakes. Wastewater from wastewater treatment plants may be contaminated with many microorganisms including *Cryptosporidium* and *Giardia* [1]. Many of these organisms are difficult to remove which is a challenge for effective wastewater treatment. When most contaminants are removed, *Cryptosporidium* and *Giardia* may be left because of their resistance to many treatment chemicals and methods [2]. Relatedly, waterborne outbreaks are always traced to many water treatment plants even after they might have met all microbiological and chemical standards for discharge [3].

The protozoan parasites, *Cryptosporidium* and *Giardia*, are known causative agents for gastrointestinal diseases both in the normal population and especially amongst immunocompromised [4]. The mode of transmission are through direct contact, contact with animals or through ingestion of contaminated water or food [5].

Both, *Cryptosporidium* oocysts and *Giardia* cysts are very resilient and may survive in water for months. In addition, they are well known to be resistant to chemical disinfection [6], which has always posed challenges for water treatment authorities, wastewater treatment plant operators and catchment authorities. Chlorination has been widely used as a disinfectant in water and wastewater treatment against many pathogens. However, for *Cryptosporidium* spp especially, the resistance against chlorine is a serious challenge in standard water treatment processes [7]. Both protozoan parasites render treatment under the South African conditions inefficient [8].

When used, low doses of chlorine are globally encouraged, since high chlorine doses may cause ecological side effects from chlorinated hydrocarbons [8]. Due to the resistance of *Cryptosporidium* to chlorine, alternative disinfectants such as chlorine dioxide, ozone and ultraviolet (UV) disinfection have been suggested and are in use in many countries [8].

UV offers an alternative for the removal of *Cryptosporidium* and *Giardia* from both water and wastewater treatment plants. The use of UV irradiation has been growing extensively in water treatment due to its demonstrated high efficiency in inactivation of *Cryptosporidium* and *Giardia* [9]. UV, as well as chlorination, treatment is normally applied as the last step in wastewater treatment, but the security and reliability normally depends on the combination of treatment steps, where the multi-barrier approach enhances the treatment robustness. The advantages of UV over chlorine as a preferred technique are inactivation efficiency, lack of disposal problems and compact with a robust design. The robust design enhances its durability in handling and transition. Some of the limitations of UV in developing countries especially include dependency on constant and secure power supply which are not mostly available in many parts of developing countries [10].

As few as 10 cysts or oocysts can cause infection. Therefore, the detection methods need to be sensitive and reliable. Effective methods of detection need to be developed for the detection of (oo)cysts in wastewater [11]. Therefore, sensitivity and reliability of methods of detection of *Giardia* and *Cryptosporidium* are important. The requirements of detection of 1 cyst per 10 to 100 liters of water are expressed for *Giardia* [12]. Flow cytometry (FCM) method for detection were recently proposed with the use of many staining methods. Most of these methods produce false-positive results [13]. While the microscopy method is laborious with many hours of laboratory experiments, flow cytometry offers a better alternative [11]. Microscopy may also be affected by mineral particles, algae and plant that may interfere with the results in environmental samples. Additionally, skilled operators are required for microscopy procedures with the need to process many samples at once [11]. Similarly, flow cytometry may also be disadvantageous in its ability to distinguish between oocysts and some autoflourescent plant, algae and mineral particles [14]. Dyes are important in assessing the viability of (oo)cysts in wastewater [15, 16]. Two fluorogenic dyes, DAPI+PI and Syto-9+PI have been used to assess the viability of *Cryptosporidium* and *Giardia* previously [17]. These dyes indicate viability through cell-wall integrity [18] and are used in this study.

In this study, different doses of chlorine and varying exposure times were used with distilled water and wastewater spiked with (oo)cysts from wastewater samples. In parallel, same samples were assessed for UV irradiation treatment efficiency at different UV doses. Microscopy and flow cytometer were used for the quantification and detection of *Cryptosporidium* and *Giardia* in wastewater. Syto-9+PI and DAPI+PI dyes were used for staining the collected wastewater samples and results were compared. This pilot experiment in this study aimed at assessing the efficiency of chlorine and ultraviolet (UV) irradiation in the inactivation of *Cryptosporidium* and *Giardia* in contaminated water.

## Methodology

In this study, different doses of chlorine and varying exposure times were used with distilled water and wastewater spiked with (oo)cysts from wastewater samples. In parallel, same samples were treated with UV irradiation treatment at different UV doses. Microscopy and flow cytometry were used for the detection of *Cryptosporidium* and *Giardia* in wastewater. Syto-9+PI and DAPI+PI dyes were used for staining the collected wastewater samples and results were compared.

### Purification of oocysts

Isolation and purification of oocysts were done from fresh raw wastewater effluent using the glucose gradient centrifugation technique and flotation in glucose-NaCl solution [19]. Samples were filtered through metal mesh-sieves 100 μm to remove debris and large particles, and later centrifuged at 3000 x g for 10 min. The sediments were suspended in PBS-Tween 80. The specific gravity of the flotation fluid was 1.07 g/mL. 300 mL of the flotation fluid was prepared by adding 50 g of glucose to 100 mL saturated NaCl solution and mixing thoroughly. 150 mL of purified water (such as Milli-Q or Super-Q) was added and mixed thoroughly until the solution was homogenous and transparent. Flotation fluid was carefully added by inserting a borosilicate glass Pasteur pipette into the same tube containing the sample so that the tip rests against the bottom of the tube. This would allow it to rise from below with a transparent layer with the sample material (mixed with PBS-Tween 80) above it. The tubes were centrifuged at 750 rpm for 10 min. Then supernatant above the layer of flotation fluid and pellet was transferred into a new tube. After that, the sample was washed by filling the tube up with purified water and then centrifuged at 3000 rpm for 10 minutes. The supernatant was aspirated down to 5 mL. The samples were then washed for two more times (3 x 10 min centrifugation in total) or more depending on how dirty the sample was. The final volume was 5 mL of purified (oo)cysts suspension that was left in the tube which was then vortexed and stored in the fridge at 4°C for future use.

### Chlorine treatment of (oo)cysts

Spiked solutions were prepared with wastewater and distilled water. The distilled water represents the control for the experiment. The water samples (both distilled water and wastewater) were chlorinated using HTH (High test hypochlorite 65% chlorine) at concentrations of 0.5 ppm, 2 ppm and 5 ppm. The chlorine and UV experiments were performed according to the methods described by Wu, Fang [20] with modifications. In a 50 mL polyethylene bottles containing the chlorinated distilled water (control) or wastewater, a portion (1 mL) of purified (oo)cysts suspension, approximately 74,600 oocysts per mL were added to each bottle and kept in the dark for adsorption at varying time intervals (15 mins, 30 mins, 60 mins and 120 mins). The samples were collected at different time intervals, and the chlorine reaction was quenched with sodium thiosulphate (Na_2_S_2_O_3_) at concentration ratio of Na_2_S_2_O_3_ to chlorine of 4:1 [21, 22]. The same amount of (oo)cysts spiked in chlorinated distilled water and wastewater was added to the controls test (untreated), which was incubated and processed the same way as the chlorine treated bottles were. This was aimed at determining the concentration of (oo)cysts that may be present in the sample (distilled water and wastewater) before spiking, that might contribute to an increase in concentration of the spiked samples, most especially in the wastewater sample. The untreated control was also run to determine the level of permeability shown by the (oo)cysts to the stains. This was to confirm that each stain is permeable to what it was supposed to stain. The pH and total dissolved solid of the spiked samples (distilled water and wastewater) were measured after filtration and before spiking with the respective (oo)cysts. The pH was 7.6 and 6.8 respectively. The total dissolved solid (TDS) was 35.5 mg/L and 250 mg/L. The wastewater used was clear to naked eye though its turbidity was not measured.

Distilled water and wastewater samples, at different chlorine concentrations (0.5 ppm, 1 ppm, 2 ppm, 5 ppm) were spiked with (oo)cysts suspension and tested at various time interval (15, 30, 60, and 120 min) for chlorine residual. Iodometric method was used for the residual chlorine test since the lowest concentration in this analysis is 0.5 ppm. However, the starch-iodide titration was used to monitor the concentration level of the chlorine during the reaction time. This was achieved by titrating sodium thiosulphate (Na_2_S_2_O_3_) against the residual chlorine concentration in the sample. The volume of Na_2_S_2_O_3_ consumed was used to calculate the concentration of chlorine in the sample with Eq (1).

$$Residual\;chlorine=\frac{volume\;of\;{Na}_{2}S_{2}O_{3}\times normality\;of\;{Na}_{2}S_{2}O_{3}\times 34.45\times 1000}{volume\;of\;sample\;taken}$$ (1)

For the UV experiments, both samples (distilled water and wastewater) were exposed to UV irradiation. A portion (1 mL) of purified (oo)cysts suspension, approximately 74,600 oocysts per mL was added to each bottle containing distilled water and wastewater. The experimental bottles used were 50 mL polyethylene bottles, round and transparent [23] with the height and diameter of 91 mm and 32 mm respectively. The surface area exposed to UV was 804 mm^2^. The experimental bottles were placed in a medium pressure mercury lamp (Heraeus GPH 212T5L/4, 10 W) and the temperature was maintained in a controlled water bath (model: D-91126, Schwabach FRG, Germany) at 25(±0.2)^o^C. The samples were collected at different time intervals of 10 seconds, 20 seconds, 40 seconds, 80 seconds and 160 seconds corresponding to the UV doses of 5.2mJ/cm^2^, 10.4 mJ/cm^2^, 20.8 mJ/cm^2^, 41.6 mJ/cm^2^ and 83.2 mJ/cm^2^ respectively. Control sample bottles were kept without UV application, incubated and processed the same way as the UV treated bottles were. The viability reduction was evaluated according to Pereira, Costa [24] by comparison between the viability of the oocysts submitted to disinfectants and oocysts control, considered 100% viable. UV doses were measured in millijoules seconds per cm^2^ (mJ/cm^2^) and were calculated using the following parameters. The relationship between these parameters can be described by the following simplified equation:

$$UV\;dose=\left(\frac{I}{UVT}\right)\;x\;t$$ (2)

Where, I = Intensity measured in milliwatts per cm^2^ (mW/cm^2^), UVT = UVTransmittance, T = Exposure time (t) (seconds)

UV dose is the product of UV light intensity and time. Dose is sometimes referred to as fluence. DOSE is equal to Intensity x Time = millijoules/(sec)(cm^2^) x time = mJ/cm^2^. Therefore, the UV fluence was calculated as in Eq (3) [25–27].

$$UV\;dose=UV\;intensity(\frac{mj}{\left(sec)({cm}^{2}\right)})\;x\;time(sec)=mJ/{cm}^{2}$$ (3)

### Viability test

The viability of the (oo)cyst after treatment with chlorine and UV was assessed and determined using vital dye Syto-9+Propidium iodide, and with the inclusion of DAPI+ Propidium iodide staining. Stained (oo)cysts were counted with flow cytometer and epifluorescence microscope. The nucleic acid dyes were supplied by Molecular Probes (Eugene, OR, U.S.A.). Live/Dead viability kit (L-34856) for flow cytometry and Live/Dead viability kit (L-7012) for microscopy and quantification assays were used. Two live/dead double-staining viability kits were considered.

### Staining procedure with Syto-9 and PI

The Live/Dead BacLight Viability assay utilizes mixtures of Syto-9 green fluorescent nucleic acid stain and the red fluorescent nucleic acid stain, propidium iodide. According to explanation given by [28], the two stains vary both in their characteristics and in their ability to enter healthy bacterial cells. The Syto-9 stain labels bacteria with both intact and damaged membranes when used alone. Whereas, propidium iodide permeates only bacteria with damaged membranes, competing with the Syto-9 stain for nucleic acid binding sites when both dyes are present. When mixed in recommended proportions, Syto-9 stain and propidium iodide produce green fluorescent staining of bacteria with intact cell membranes and red fluorescent staining of bacteria with damaged membranes [28].

Viability of the (oo)cysts were determined by double-staining of (oo)cysts with the use of Live/Dead BacLight kit (Invitrogen) was performed according to [29] adapted from [30] with little modification. The Live/Dead BacLight kit was removed from -20°C light protected storage and allowed to thaw at room temperature. Equal volumes of the two reagents (Syto-9 and propidium iodide) were combined in a 1.5 mL Eppendorf tube and mixed. The tube was wrapped with aluminium foil in order to avoid light penetration into the tube containing the staining solution. Three microliters of this mixture were then added to 97μL of DW, containing 2 × 10^4^ oocysts. After incubation at 37°C for 60 min, 10 μL of aliquots were placed on a slide and microscopical observations were made. Samples were observed under Axiolab Zeiss fluorescence microscope, using either 400× or 1000× magnification. Colour and intensity of live (dark green) and dead (bright red or orange/yellow) (oo)cysts staining were visually assessed. Another aliquot from staining solution 100 μL was in the fluidic sample tube and analysed with flow cytometer. A green filter block was used to examine the Syto-9 and PI (propidium iodide) stained (oo)cysts. Nonviable (oo)cysts were fluorescent red, while viable (oo)cysts were fluorescent green.

### Reagent quality and performance check of the fluorogenic dyes

Method performance of the dyes were checked to confirm if the specific dye was staining what they were meant to stain and penetrate into. This was to assess the usefulness of these dyes as indicators of viability for *Cryptosporidium* oocysts and *Giardia* cysts. These stains vary both in their spectral characteristics and in their ability to penetrate into the cells. Therefore, samples were also analysed separately for each stain (DAPI only, PI only and Syto-9 ONLY) along with all samples analysed with DAPI+PI and Syto-9 + PI. This was performed to compare and to establish performance criteria of each stain in order to determine if the results of analyses from each stain was correct.

### Staining procedure DAPI and PI

Incubation of oocysts with DAPI and PI was performed according to the protocols described by [15]. Working solutions of DAPI (2 mg/mL in absolute methanol) and PI (1 mg/mL in 0.1 M PBS, pH 7.2) were prepared and stored at 4°C in the dark. 100 μl of test suspension containing approximately 60.1 X 10^3^ to 74.6 X 10^3^ were incubated simultaneously with 10 μl of DAPI working solution and 10 μl of PI working solution at 37°C for 2h. (oo)cysts were viewed by fluorescence microscopy and also analysed by flow cytometry.

## Microscopy

Ten-microliter aliquots of oocyst suspension was viewed under epifluorescence microscope equipped with a UV filter block (365-nm excitation, 445-nm emission) for DAPI and a green filter block (500-nm excitation, 630-nm emission) for PI. Images were captured using a Zeiss AxioCam MRc (Carl Zeiss, Germany) camera and image quantification was carried out using the Zeiss AxioVision Release 4.6 (12–2006) imaging software. Proportions of ruptured (ghost), PI-positive (PI+), DAPI positive/PI positive (DAPI+PI+), DAPI-positive/PI-negative (DAPI+ PI-), DAPI negative PI-negative (DAPI- PI-) oocysts were quantified in each sample.

An epifluorescence microscope was used at either 400× or 1000× magnification to examine all samples. *Cryptosporidium* oocysts were defined as being spherical in shape, 4–7 μm in diameter, with a surface fold sometimes visible, highly retractile with phase contrast, and with sporozoites or cytoplasm visible with DIC. *Giardia* cysts were defined as oval or spherical in shape, 6–16 μm in diameter, highly retractile with phase contrast, with diagnostic internal structures (trophozoite nucleii, etcetera) [11].

## Flow cytometry

The flow cytometry analysis was performed on FACSCalibur BD Biosciences, Sydney standard model, with three PMTs equipped with standard filters (FL1: LP 695/40 nm; FL2: LP 585/42 nm; FL3: LP 488/10 nm; FL4: LP 780/60 nm; FL5: LP 616/23 nm FL6: LP 530/30 nm). The machine was fitted with an argon ion laser operating at 488 nm Argon Laser and with cell Quest Pro software (version 4.0.2, BD Biosciences, Sydney). Acquisition settings were defined using a no stained sample (autofluorescence), adjusting the PMTs voltage to the first logarithmic (log) decade. Instrument controls followed standard procedures adapted from [31]. Sheath fluid consisted of 2.0 mM potassium phosphate buffer (pH 6.8). The detectors used were forward angle light scatter (FALS) and side-angle light scatter (SALS).

The instruments were calibrated daily with Coulter ImmunoCheck fluorescent beads, according to the manufacturer's instructions. The laser output, high voltage, and gains of the detectors were adjusted to obtain the tightest populations of purified oocysts and cysts with maximum separation from background debris in samples. To determine what was represented by each population that appeared on the Log FSC vs Log SSC scatter-plot, cell sorting was performed, and samples of each population sorted on to microscope slides were examined microscopically [11].

### Data analysis

Percentages of *Cryptosporidium* and *Giardia* viabilities were calculated for the UV and chlorine exposures at different exposure times and doses for all the trials. The mean and standard deviation of percentage viability of *Cryptosporidium* and *Giardia* after exposure to UV radiation and chlorine were also calculated and reported. In this study, descriptive analysis was done on the percentage viability to determine the mean and standard deviation of *Cryptosporidium* and *Giardia* in distilled water and wastewater. This was to assess the relationship between the mean percentage viability between the different doses of chlorine and UV on both distilled water and wastewater. The two dyes were compared graphically. This was to determine the effect of different dyes on the results of mean percentage viability. The relationship between the methods of detection, viz cytometry and microscopy, were also compared with the use of graphs.

## Results and discussion

Effects of UV on viability at different doses are presented and discussed in section (a) while section (b) presents the effect of different doses of chlorine and duration of exposure on viability. In both sections, comparison between UV and chlorination are presented and discussed. In section (c), effect of turbidity on viability and comparison between the results obtained using the two different dyes, Syto-9+PI and DAPI+PI, are presented and discussed. Finally, Section (d) presents the comparison between microscopy and flow cytometry results during the experiment.

### Effect of UV on viability

**Fig 1:**
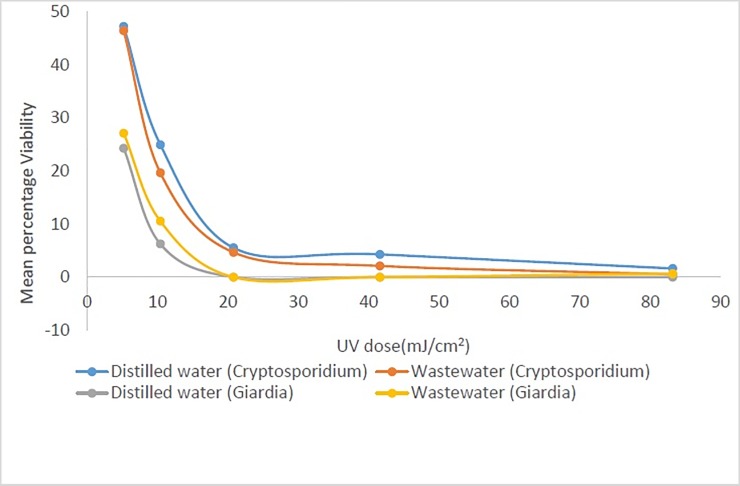
Mean percentage viability of *Cryptosporidium* and *Giardia* related to UV doses in treatment with spiked samples in distilled water and wastewater samples.

**Fig 2:**
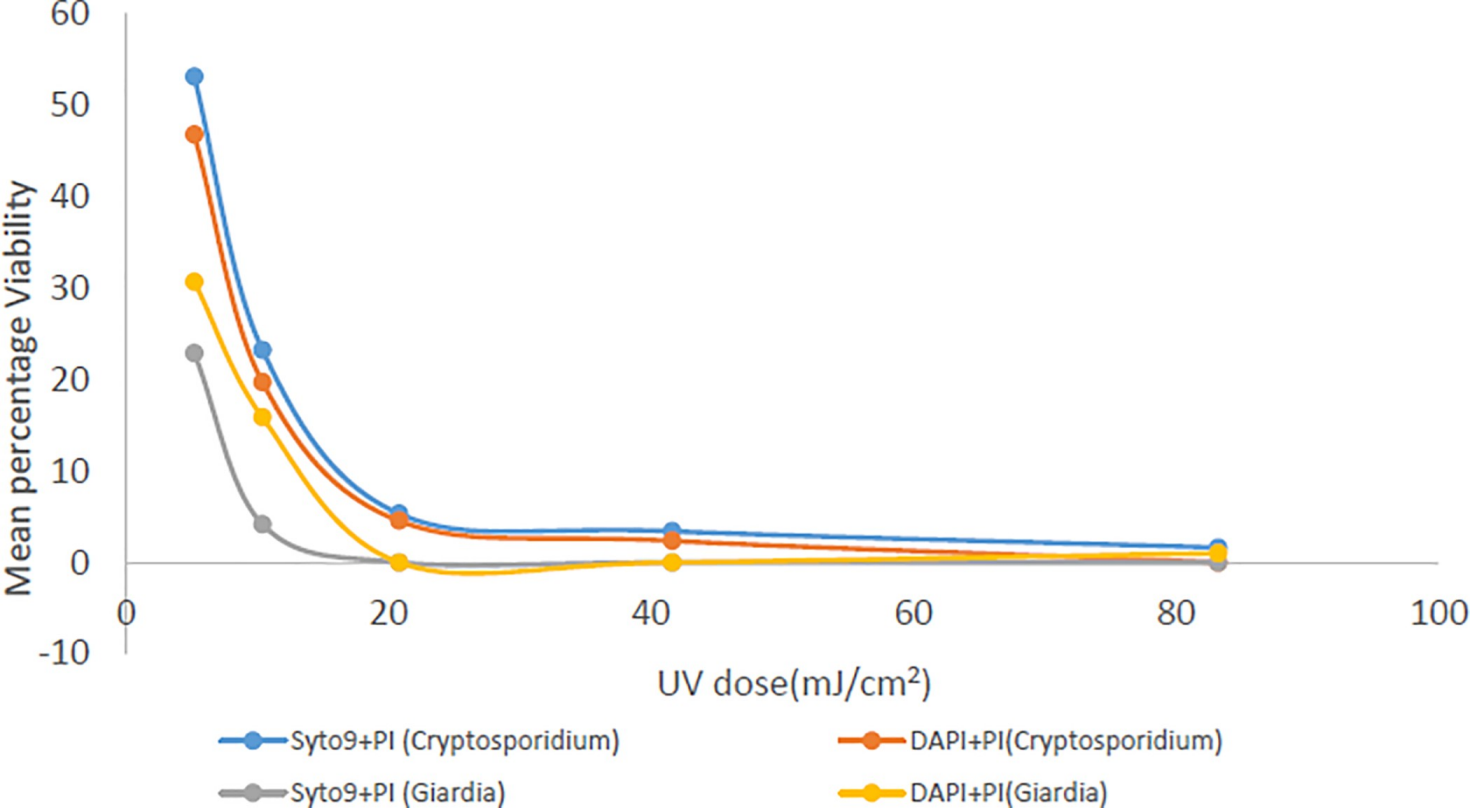
Mean percentage viability of *Cryptosporidium* and *Giardia* at different UV doses using Syto-9+PI and DAPI+PI dyes.

Table 1 presents the effect of the UV dose on the die-off of *Giardia* cysts and *Cryptosporidium* oocysts as measured with microscopic counts using DAPI+PI and Syto-9+PI. Table 2 presents the same effect as measured with flow cytometry counts using same dyes (DAPI+PI and Syto-9+PI). The comparative impacts on the die-off in distilled water versus in wastewater are further presented in Fig 1 and the effect due to different stains in Fig 2. Overall, the UV efficiency is higher in *Giardia* than in *Cryptosporidium* but with a common cut-off value of 20 mJ/cm^2^ based on selected doses (Table 1). [32] reported that UV irradiation usually inactivates *Cryptosporidium* by attacking the nucleic acid and thereby preventing multiplication of the parasite. *Giardia* is thus less resistant to UV irradiations than *Cryptosporidium* in line with the conclusion by [33] that *Giardia* responded very well to UV irradiation. It can be observed that UV irradiation has more effect on *Giardia* than chlorine (Table 1). This is due to the killing effect exhibited by UV on *Giardia* because of its effect on its DNA [33]. The same effect relates to *Giardia* destruction [33]. Die-off of the (oo)cysts is directly proportional to the increase in exposure time and the UV doses [34]. The sensitivity of *Giardia* to UV irradiation was higher than *Cryptosporidium* as found in the obtained results and was also observed by [33]. As a support to the obtained results in the present study, Campbell and Wallis [35] showed that UV irradiation at 20 to 40 mJ/cm^2^ is capable of killing 99.9% of *Giardia* cysts. Rahdar and Daylami [33] further proved that UV radiation of 50mJ/cm^2^ at 280nm can destroy only *Giardia* cysts from feaces of infected patients to a maximum of 75%. However, the results in this present study are slightly different from this. The test regime of this study, where (oo)cysts were extracted from environmental water samples, was different from those studies [33, 35]. This may be accountable for the different results generated in this present experiment.

**Table 1:** Mean and SD of viability (%) after exposure to chlorine and UV irradiation using microscopy.

Number of Exposure time	Duration of Exposure(min)	Chlorine dose			Number of Exposure time	UV	
		0.5 ppm	2 ppm	5 ppm		UV dose (mJ/cm^2^)	Mean±SD
	*Cryptosporidium*						
1	15	86±2	80±6	42±8	1	5.2	47±5
2	30	70±6	67±4	32±2	2	10.4	22±7
3	60	55±6	59±5	23±2	3	20.8	5±1
4	120	46±4	50±6	15±3	4	41.6	3±2
					5	83.2	1±1
	*Giardia*						
1	15	57±32	7±0	7±0	1	5.2	25±7
2	30	30±21	0±0	0±0	2	10.4	7±9
3	60	0±0	0±0	0±0	3	20.8	0±0
4	120	0±0	0±0	0±0	4	41.6	0±0
					5	83.2	0±0

**Table 2:** Mean and SD of viability (%) of (oo)cysts after exposure to chlorine and UV irradiation using flow cytometry.

Number of Exposure time		Chlorine dose			Number of Exposure time	UV	
	Duration of Exposure (min)	0.5 ppm	2 ppm	5 ppm		UV dose (mJ/cm^2^)	
1	15	82±3	1	41±1	1	5.2	43±1
2	30	79±1	2	33±1	2	10.4	12±1
3	60	71±1	3	21±1	3	20.8	10±0
4	120	66±3	4	12±0	4	41.6	8±0
					5	83.2	3±0

It was reported by [36] that *Cryptosporidium* 2 log excystation reduction requires high UV dose of 230mWs/cm^2^. This was the recommended dose for the purified water with fresh faecal samples used for their experiment. They further explained that the UV dose required for 1-, 2-, and 4-log reduction in infectivity in the purified water with fresh faecal samples were 0.48, 0.97 and 1.92 mWs/cm^2^ respectively which are higher than the doses used in the present study. Moreover, a 200 times higher dose will be required for a 2-log reduction in viability than reduction in infectivity as assessed by *in vitro* excystation. This demonstrates that if *Cryptosporidium* oocysts are exposed to low dose of UV irradiation, they may not be infectious but they will still be able to excyst in the purified water with faecal samples [36].

In Table 3, the mean percentages and standard deviation of viable *Cryptosporidium* and *Giardia* at different UV doses gave a remaining 1±1% of viable *Cryptosporidium* at 83.2 mJ/m^2^. At 20.8 mJ/m^2^, all *Giardia* were already dead. This further confirms that higher UV doses are required to eliminate *Cryptosporidium* than *Giardia*. Prolonged UV exposure could also be more potent than short exposure as reported by [34]. Moreover, at low UV doses, a fraction of viable *Cryptosporidium* and *Giardia* are affected as found in Table 1, where a remaining 47% and 25% of *Cryptosporidium* and *Giardia* were counted as viable respectively at 5.2 mJ/cm^2^ dose. It can further be established that UV irradiations have more effect on *Giardia* than *Cryptosporidium* in all the trials as reported (Table 1; Figs 1 and 2). *Cryptosporidium* reduction in viability due to UV exposure in wastewater is encouraging and also in agreement with [37] who found that solar UV can rapidly inactivate *Cryptosporidium* in environmental waters.

**Table 3:** Mean percentages of viable *Cryptosporidium* and *Giardia* for different UV doses using microscopy.

Trials	UV dose (mJ/cm^2^)									
	5.2 mJ/cm^2^		10.4 mJ/cm^2^		20.8 mJ/cm^2^		41.6 mJ/cm^2^		83.2 mJ/cm^2^	
	*Cryptosporidium* *(%)*	*Giardia* *(%)*	*Cryptosporidium* *(%)*	*Giardia* *(%)*	*Cryptosporidium* *(%)*	*Giardia* *(%)*	*Cryptosporidium* *(%)*	*Giardia* *(%)*	*Cryptosporidium* *(%)*	*Giardia* *(%)*
1	53	20	33	0	6	0	6	0	2	0
2	41	29	17	13	5	0	2	0	1	0
3	46	20	20	0	5	0	2	0	2	0
4	47	33	20	17	5	0	2	0	0	0
5	46	28	19	15	4	0	3	0	0	2
Mean±SD										
Distilled water	47.0±8.5	24.5±6.4	25.0±11.3	6.5±9.2	5.5±0.7	0.0±0.0	4.0±2.8	0.0±0.0	1.5±0.7	0.0±0.0
Wastewater	46.3±0.6	27.0±6.6	19.7±0.6	10.7±9.3	4.7±0.6	0.0±0.0	2.3±0.6	0.0±0.0	0.7±1.2	0.7±1.2
Syto-9+PI	46.7±6.0	23.0±5.2	23.3±8.5	4.3±7.5	5.3±0.6	0.0±0.0	3.3±2.3	0.0±0.0	1.7±0.6	0.0±0.0
DAPI+PI	46.5±0.7	30.5±3.5	19.5±0.7	16.0±1.4	4.5±0.7	0.0±0.0	2.5±0.7	0.0±0.0	0.0±0.0	1.0±1.4

Note: Trials 1 to 2: Distilled water. Trials 3 to 5: Wastewater. Trials 1 to 3 were performed using Syto-9+PI stains and Trials 4 to 5 were performed using DAPI+PI stains.

Fig 1 illustrates the reduction in mean percentage viability of *Cryptosporidium* and *Giardia* as related to treatment with different UV doses. The results presented in Fig 2 represent the mean percent viability using both dyes for the 5 trials (that is Syto-9+PI in trials 1 to 3 and DAPI+PI in trials 4 to 5). All *Giardia* originally present were eliminated at 20.8 mJ/cm^2^ of UV dose which confer its efficiency in removing *Giardia* from wastewater. Less than 2% of *Cryptosporidium* was viable after 83 mJ/cm^2^ UV exposure. An increase in exposure dose can significantly reduce the viability of both *Cryptosporidium* and *Giardia* in water. It was found in this study that UV disinfection was much more effective than chlorination. This same conclusion was noted by [38].

### Effect of chlorination on viability

**Fig 3:**
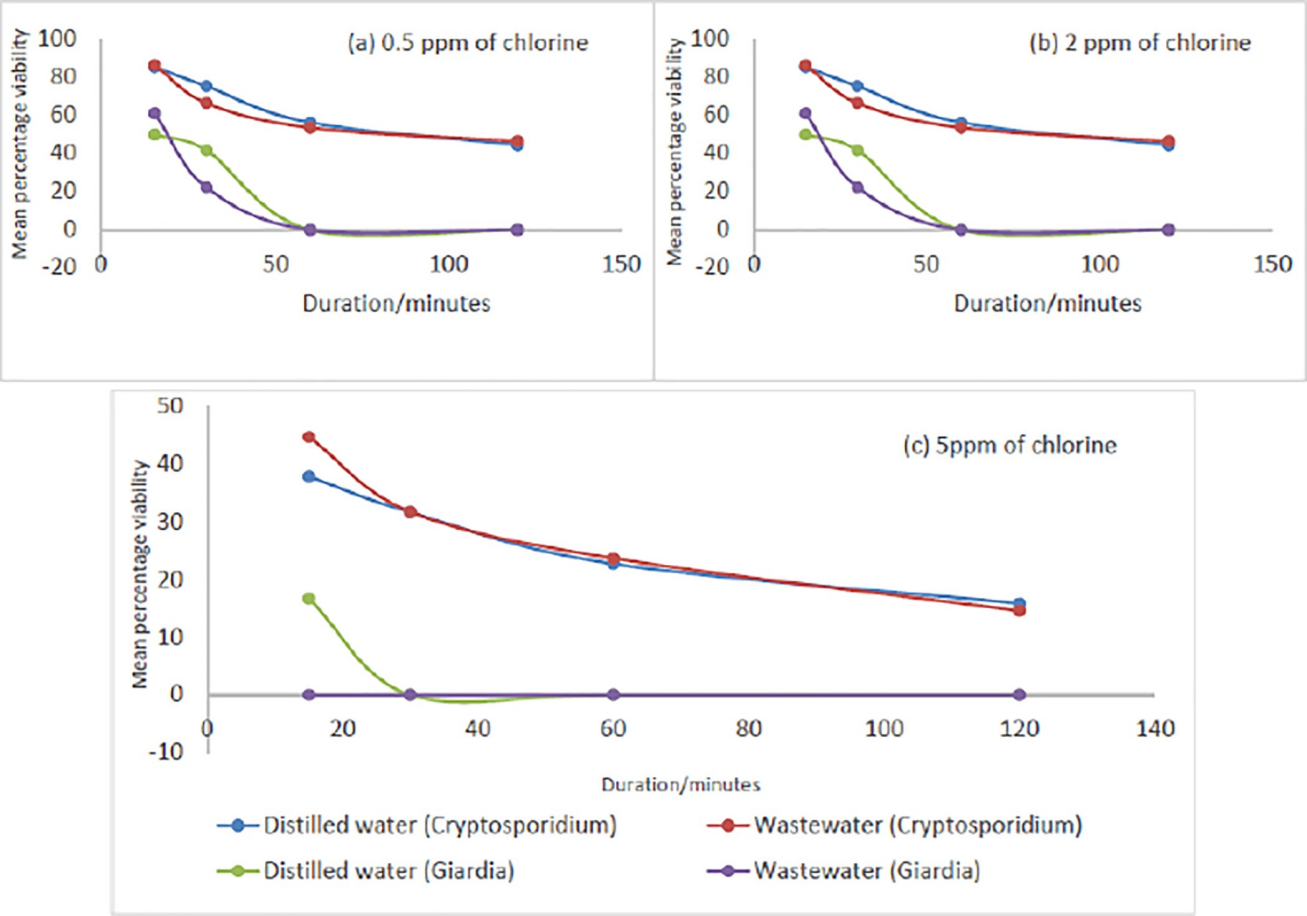
Mean percentage viability of *Cryptosporidium* and *Giardia* after prolonged exposure to 0.5 ppm, 2 ppm and 5 ppm of chlorine treatments for distilled water and wastewater.

Table 4 presents the mean percentages of viable *Cryptosporidium* and *Giardia* after exposure to 0.5 ppm, 2 ppm and 5 ppm of chlorine during different exposure times. For 0.5 ppm, *Giardia* became non-viable after 60 minutes of exposure except in trial 4 where they are non-viable after 30 minutes. Half of the *Cryptosporidium* were still viable (42 to 47% for distilled water and 42 to 51% for wastewater) after 120 minutes exposure time. The findings of [33] supports this finding that lower concentration of chlorine results in the elimination of small amount of oocysts. Since the reduction in distilled water and in wastewater were similar, the data has been treated jointly. Thus, the mean viable fraction of *Cryptosporidium* decreased from 86±2% after 15 minutes’ exposure to 0.5 ppm of chlorine to 46±4% viability after 120 minutes. However, 0.5 ppm of chlorine was not judged as effective for *Cryptosporidium* removal even after long exposure time (120 minutes) when compared to the effect on *Giardia* in wastewater (Fig 3). A low dose of chlorine (0.5 ppm) with about 60 minutes exposure time may be sufficient for *Giardia* removal as all *Giardia* present were non-viable after 60 minutes. These results were corroborated in the World Health Organisation study [39] that reported that *Giardia* is less resistant to disinfection including chlorination. In Table 4, at 2 ppm, it was found that all *Giardia* became destroyed after 15 minutes in all the 4 trials (2 to 5) except in trial 1 where *Giardia* became destroyed after 30 minutes. It can be found that the same number of *Giardia* was destroyed with chlorine dose of 0.5 ppm (60 minutes exposure time) and 2 ppm (15 minutes exposure time). The effectiveness can thus be either enhanced by increased exposure time or through increase in dose [40].

**Table 4:** Mean percentages of viable *Cryptosporidium* and *Giardia* for different chlorine exposures at different durations using microscopy.

0.5 ppm								
DURATION /min	15		30		60		120	
Trials	*Cryptosporidium*	*Giardia*	*Cryptosporidium*	*Giardia*	*Cryptosporidium*	*Giardia*	*Cryptosporidium*	*Giardia*
1	84	67	77	33	53	0	42	0
2	87	33	74	50	60	0	47	0
3	84	33	69	33	47	0	42	0
4	89	100	71	0	58	0	51	0
5	87	50	59	33	55	0	46	0
Mean±SD								
Distilled water	85.5±2.1	50.0±24.0	75.5±2.1	41.5±12.0	56.5±4.9	0.0±0.0	44.5±3.5	0.0±0.0
Wastewater	86.7±2.5	61.0±34.8	66.3±6.4	22.0±19.1	53.3±5.7	0.0±0.0	46.3±4.5	0.0±0.0
Syto-9+PI	85.0±1.7	44.3±19.6	73.3±4.0	38.7±9.8	53.3±6.5	0.0±0.0	43.7±2.9	0.0±0.0
DAPI+PI	88.0±1.4	75.0±35.4	65.0±8.5	16.5±23.3	56.5±2.1	0.0±0.0	48.5±3.5	0.0±0.0
2 ppm								
1	81	33	66	0	59	0	48	0
2	79	0	72	0	63	0	54	0
3	73	0	64	0	54	0	46	0
4	79	0	71	0	64	0	57	0
5	87	0	64	0	55	0	46	0
Mean±SD								
Distilled water	80.0±1.4	16.5±23.3	69.0±4.2	0.0±0.0	61.0±2.8	0.0±0.0	51.0±4.2	0.0±0.0
Wastewater	79.7±7.0	0.0±0.0	66.3±4.0	0.0±0.0	57.7±5.5	0.0±0.0	49.7±6.4	0.0±0.0
Syto-9+PI	77.7±4.2	11.0±19.1	67.3±4.2	0.0±0.0	58.7±4.5	0.0±0.0	49.3±4.2	0.0±0.0
DAPI+PI	83.0±5.7	0.0±0.0	67.5±4.9	0.0±0.0	59.5±6.4	0.0±0.0	51.5±7.8	0.0±0.0
5 ppm								
1	42	33	34	0	25	0	15	0
2	33	0	29	0	21	0	17	0
3	44	0	34	0	25	0	12	0
4	39	0	30	0	21	0	14	0
5	51	0	31	0	25	0	18	0
Mean±SD								
Distilled water	37.5±6.4	16.5±23.3	31.5±3.5	0.0±0.0	23.0±2.8	0.0±0.0	16.0±1.4	0.0±0.0
Wastewater	44.7±6.0	0.0±0.0	31.7±2.1	0.0±0.0	23.7±2.3	0.0±0.0	14.7±3.1	0.0±0.0
Syto-9+PI	39.7±5.9	11.0±19.1	32.3±2.9	0.0±0.0	23.7±2.3	0.0±0.0	14.7±2.5	0.0±0.0
DAPI+PI	45.0±8.5	0.0±0.0	30.5±0.7	0.0±0.0	23.0±2.8	0.0±0.0	16.0±2.8	0.0±0.0

Note: Trials 1 to 2: Distilled water. Trials 3 to 5: Wastewater. Trials 1 to 3 were performed using Syto-9+PI stains and Trials 4 to 5 were performed using DAPI+PI stains.

*Cryptosporidium* is smaller and more robust than *Giardia* which makes it to withstand the chlorine doses that are used in the water treatment processes. Their resistance has caused several water borne outbreaks worldwide [7, 41–43]. Water authorities need to combine multiple treatment barriers for efficient *Cryptosporidium* removal in wastewater [16].

**Fig 4:**
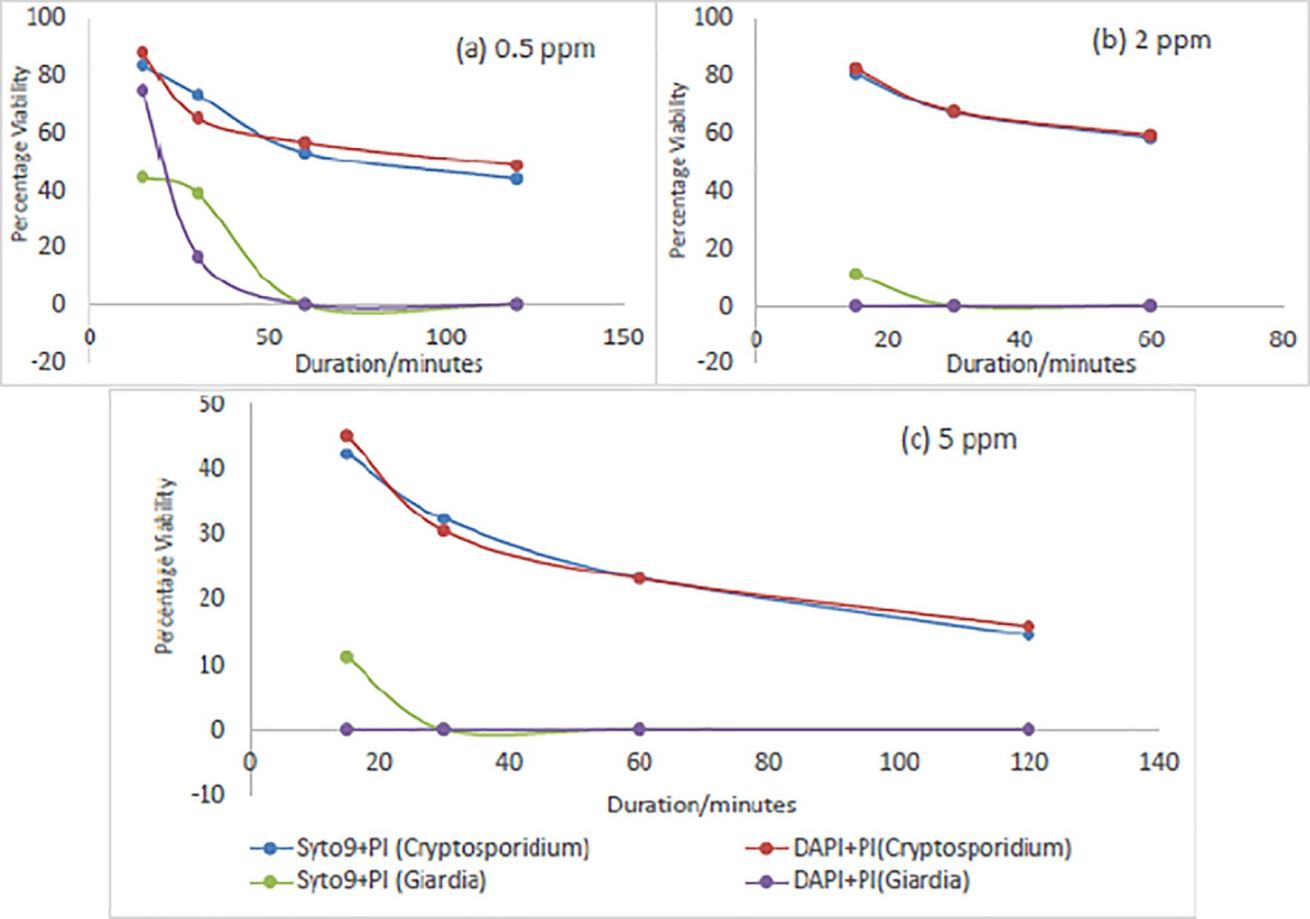
Mean percentage viability of *Cryptosporidium* and *Giardia* at different chlorine exposures using Syto-9+PI and DAPI+PI dyes for distilled water and wastewater (microscopy method).

The results presented in Fig 4 compares the mean percentage viability using direct microscopy with Syto-9+PI in trials 1 to 3 and DAPI+PI in trials 4 to 5. In Fig 4(C), there is a steady reduction in viability of *Giardia* until after 30 minutes when all *Giardia* were dead for 5 ppm. Therefore, increasing the duration of exposure may not really influence the viability of *Giardia* as found after 50 minutes for both 0.5 ppm and 2 ppm (Figs 4(A) and 4(B)) and 30 minutes for 5 ppm (Fig 4(C)). For *Cryptosporidium*, a slight reduction in viability was invariably noticed after 100 minutes for 5 ppm (Fig 4(C)) and after 50 minutes for 0.5 ppm and 2 ppm (Fig 4(B) and 4(C)). In conclusion, an increase in exposure time may be significant up to a threshold, in this case, at 100 minutes for 5 ppm and 50 minutes for 0.5 ppm and 2 ppm, after which increase in exposure time may not have any appreciable reduction in viability. There were no significant trends as depicted by Fig 4. This further confirms that increasing chlorine dose from 0.5 ppm to 2 ppm may not have effect on the viability of *Cryptosporidium* unlike an appreciable reduction with the viability of *Giardia*. This points out that *Cryptosporidium* is more resistant to chlorine than *Giardia* when exposed under the same environmental conditions [16, 44, 45].

All *Giardia* present were eliminated after 15 minutes exposure time for 5 ppm which is similar to 2 ppm dose (Table 4). Increasing chlorine dose from 2 ppm to 5 ppm, makes no difference to *Giardia* viability. It shows that it is a waste of resources to increase chlorine dose from 2 ppm to 5 ppm for the sole aim of *Giardia* removal in wastewater. There was appreciable viability reduction for *Cryptosporidium* with chlorine dose increase from 2 ppm to 5 ppm but no reduction in *Giardia* for same increase (Table 4). More than half of *Cryptosporidium* was destroyed after 15 minutes exposure with 5 ppm (42±8% viability). Similarly, after 120 minutes of exposure with 5 ppm dose, almost 90% of *Cryptosporidium* was eliminated (15±3% viability). This shows that increasing the chlorine dose to 5 ppm has significant effect on the reduction in viability of *Cryptosporidium* with almost total elimination. However, chlorine may not remove all *Cryptosporidium* present in wastewater as there was no significant reduction in viability after 100 minutes of exposure. Fig 3(C) shows that mean percentage viability is inversely proportional to duration of exposure with a sharp decrease in mean percentage viability from 15seconds to 80 minutes and slight decrease from 100 minutes to 120 minutes showing that an increase in exposure duration has limited effect at 100 minutes to 120 minutes and beyond. Additionally, it can be inferred that both duration of exposure and dose have effect on viability for both *Cryptosporidium* and *Giardia* but at different stages [44]. The effect of increase in dosage is more pronounced at the beginning of experiment where there are sharp decreases in viability.

At a low dose, duration of exposure may need to be increased for a reduction in viability. This will ensure that more *Cryptosporidium* and *Giardia* are eliminated over a longer time with slow reaction with chlorine. In summary, an increased dose is more effective than exposure duration. This assertion can be supported with findings in Table 4 which presents the mean and standard deviation of viability of *Cryptosporidium* and *Giardia* after exposure to chlorine. At 15 minutes, about 86±2%, 80±6% and 42±8% of *Cryptosporidium* was still viable at 0.5 ppm, 2 and 5 ppm respectively. The comparative values at 120 minutes were 46±4%, 50±6% and 15±3% of *Cryptosporidium* were viable showing the effect of duration of exposure and increase in chlorine dose. The same phenomenon was observed for *Giardia* where increase in dose from 0.5 ppm to 2 ppm reduced viability from 57±32% to 7±0% at 15 minutes. However, further increment from 2 ppm to 5 ppm did not decrease viability as it remained at 7±0%. Increasing the duration of exposure from 15 minutes to 30 minutes has a pronounced effect on to viability as it decreased to 30±21% from 57±32% for 0.5 ppm and 0% from 7±0% for 2 ppm and 5 ppm. Further increase in duration of exposure from 30 minutes to 60 minutes and later to 120 minutes did not decrease viability of *Giardia* as all *Giardia* present were non-viable after 30 minutes (Table 5).

**Table 5:** Comparison between the percentages of viability of (oo)cysts when using microscopy and flow cytometer for UV irradiations.

UV dose (mJ/cm^2^)	5.2		10.4		20.8		41.6		83.2	
Trials	Microscopy	Flow cytometer	Microscopy	Flow cytometer	Microscopy	Flow cytometer	Microscopy	Flow cytometer	Microscopy	Flow cytometer
1	50	43	30	12	6	9	6	8	2	3
2	40	43	17	12	4	10	2	8	1	3
3	44	45	18	12	5	10	2	9	2	3
4	46	43	20	11	5	10	2	9	0	3
5	43	45	19	12	5	10	5	9	2	3
**Mean±SD**	**45±4**	**44±1**	**21±5**	**12±1**	**5±0**	**10±0**	**3±2**	**8±0**	**1±1**	**3±0**

Note: Trials 1 to 2: Distilled water. Trials 3 to 5: Wastewater. Trials 1 to 3 were performed using Syto-9+PI stains and Trials 4 to 5 were performed using DAPI+PI stains.

The same trend was observed for UV exposure in Table 3. After 10.4mJ/cm^2^, all *Giardia* present became dead. Further increase in dose became unnecessary after 10.4 mJ/cm^2^. Conversely, there was a slight decrease in the viability of *Cryptosporidium* after 20.8 mJ/cm^2^ from 5±0% to 3±1% and later to 1±1% after 20.8 mJ/cm^2^, 41.6 mJ/cm^2^ and 83.2 mJ/cm^2^ respectively unlike sharp decrease experienced after 5.2 mJ/cm^2^ (100% to 47±3%), 10.4 mJ/cm^2^ (4.7±3% to 22±1%) and 20.8mJ/cm^2^ (22±1% to 5±0%). In spite of this, Table 5 shows that UV is more effective in removing *Cryptosporidium* and *Giardia* in wastewater than chlorine even at higher doses of chlorine. While chlorine at 5 ppm has 15±3% viability even at 120 minutes, UV has 3±1% viability at 41.6 mJ/cm^2^(80 seconds). This agrees with previous studies where efficacy of UV over chlorine has been demonstrated in the removal of *Cryptosporidium* and *Giardia* in wastewater [9, 36, 37, 46]. Chlorine at higher doses may not be recommended due to the toxic by-products such as trihalomethanes (THMs) [47]. Higher doses above 2 ppm concentrations are not used in conventional water treatment and also not economically viable in relation to water treatment stations [24].

### Effect of turbidity

When comparing the results between the distilled water and the wastewater experiment, a slightly higher fraction of viable (oo)cysts were obtained in the wastewater samples especially in chlorine treatment (Tables 3 and 4). The turbidity would affect the water treatment efficiency using UV irradiation as depicted in Table 3 when both *Cryptosporidium* and *Giardia* are considered together. The mean percentage viabilities for distilled water and wastewater for all UV exposures were comparable for *Cryptosporidium*. This shows that viability was not really affected by opacity for *Cryptosporidium* removal. For *Giardia*, higher values were recorded for wastewater in many of the trials showing that higher turbidity causes increase in viability due to UV opacity. The mean percentage viability for distilled water (Trials 1 and 2) and wastewater (Trials 3 to 5) in Table 4 are however comparable with values for wastewater slightly higher at lower exposure durations (15 to 30 minutes) especially for *Giardia*. Turbidity, thus, slightly affected the treatment efficiency, especially when low doses of chlorine were applied. *Cryptosporidium* percentage viability for distilled water and wastewater were comparable. Water with low turbidity responds better to chlorine treatment than turbid water (wastewater).

### Comparison between microscopy and flow cytometry

When assessing the results obtained using microscopy as compared to flow cytometry for UV irradiations in Table 5, the results for both methods showed decreasing viability with increasing irradiation where microscopy gave slightly higher viability. It was also evident that percentages of viability were presented for both microscopy and flow cytometer (Table 6). It can be observed further that microscopy has higher mean percentage viability value at 15 minutes while flow cytometry has higher percentage values at 30 minutes, 60 minutes and 120 minutes for 0.5 ppm. This shows that flow cytometry gives higher mean percentage viability values than microscopy especially at 0.5 ppm chlorination for both distilled water and wastewater. Tables 4 and 5 at Trials 2 and 3 shows that *Cryptosporidium* and *Giardia* have higher mean percentage viability values at Trial 2 (distilled water) than Trial 3 (wastewater) using the same dye (Syto-9+PI) for staining. This shows that under the same condition and staining, chlorination may have lower effect on wastewater than distilled water. In this experiment, turbidity did not have high significance on the treatment efficiency in all the trials. This may be due to the type of stains used.

**Table 6:** Comparison between the percentages of viability of (oo)cysts when using microscopy and flow cytometer for chlorine exposures.

	**0.5 ppm**							
DURATION /min	15		30		60		120	
Trials	Microscopy	flow cytometer	Microscopy	flow cytometer	Microscopy	flow cytometer	Microscopy	flow cytometer
1	83	85	75	76	51	71	40	67
2	85	82	73	80	58	72	46	67
3	82	77	68	81	45	70	41	63
4	89	83	70	79	57	69	50	69
5	86	81	58	78	54	72	45	66
**MEAN±SD**	**85±3**	**82±3**	**69±6**	**79±1**	**53±6**	**71±1**	**44±4**	**66±3**
**2 ppm**								
1	79	85	63	76	57	71	46	67
2	77	82	70	80	61	72	53	67
3	70	77	61	81	52	70	44	63
4	77	83	70	79	63	69	54	69
5	84	81	62	78	54	72	45	66
**MEAN±SD**	**77±6**	**82±3**	**65±5**	**79±1**	**57±5**	**71±1**	**48±5**	**66±3**
**5 ppm**								
1	42	40	33	31	24	21	14	12
2	32	42	28	32	20	21	16	13
3	42	41	33	33	24	21	11	12
4	39	42	30	35	21	21	14	13
5	50	42	30	33	24	23	17	13
**MEAN±SD**	**41±7**	**41±1**	**31±2**	**33±1**	**23±2**	**21±1**	**15±3**	**12±0**

Note: Trials 1 to 2: Distilled water. Trials 3 to 5: Wastewater. Trials 1 to 3 were performed using Syto-9+PI stains and Trials 4 to 5 were performed using DAPI+PI stains.

**Fig 5:**
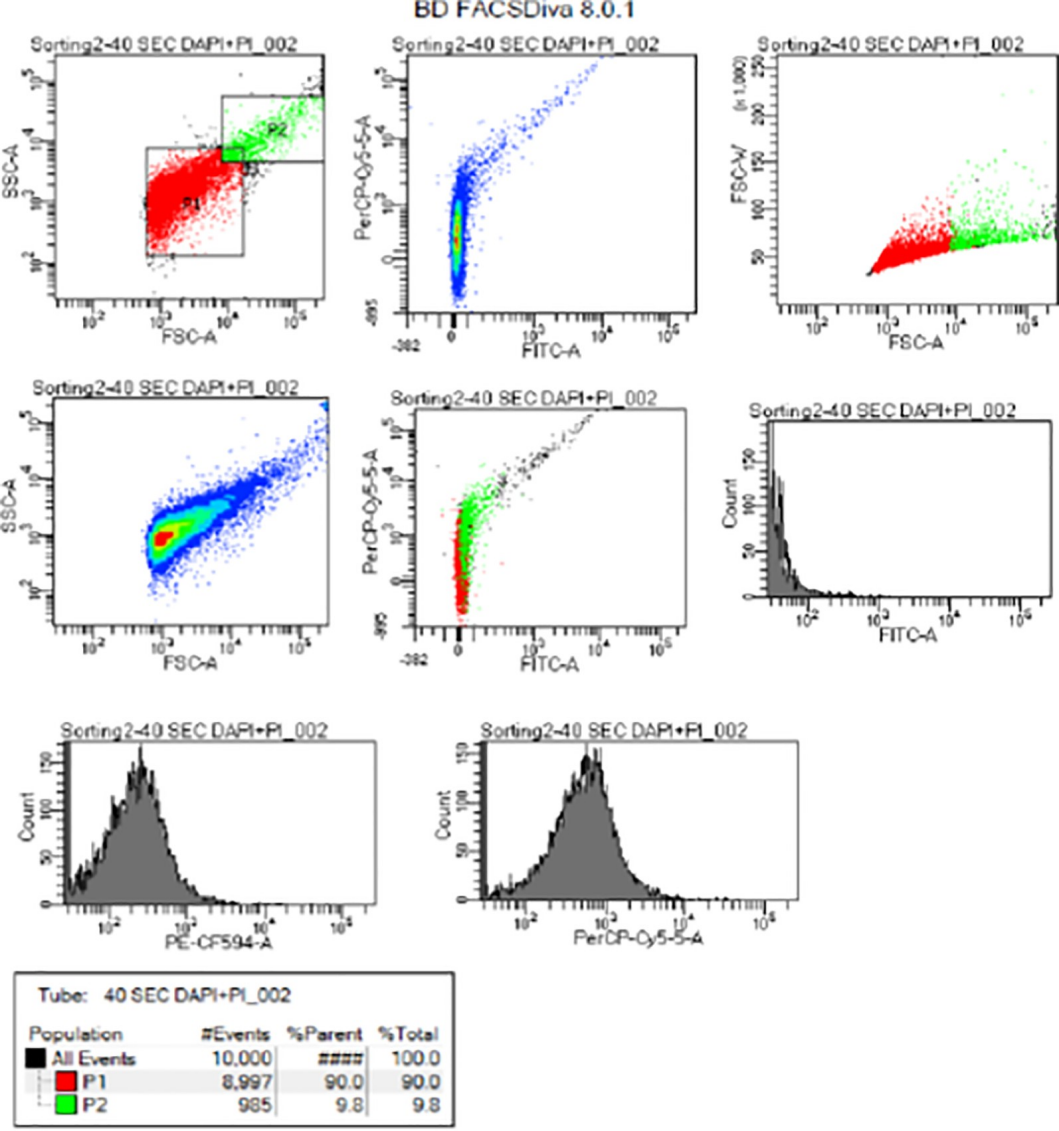
Flow cytometry histogram and dot plots showing viability of *Cryptosporidium* and *Giardia* at 40 seconds after exposure to UV irradiation. Note: Samples of Region P1 and P2 represent gates for non-viable and viable (oo)cysts with P1 as side-scatter (SSC) against forward side-scatter (FSC).

**Fig 6:**
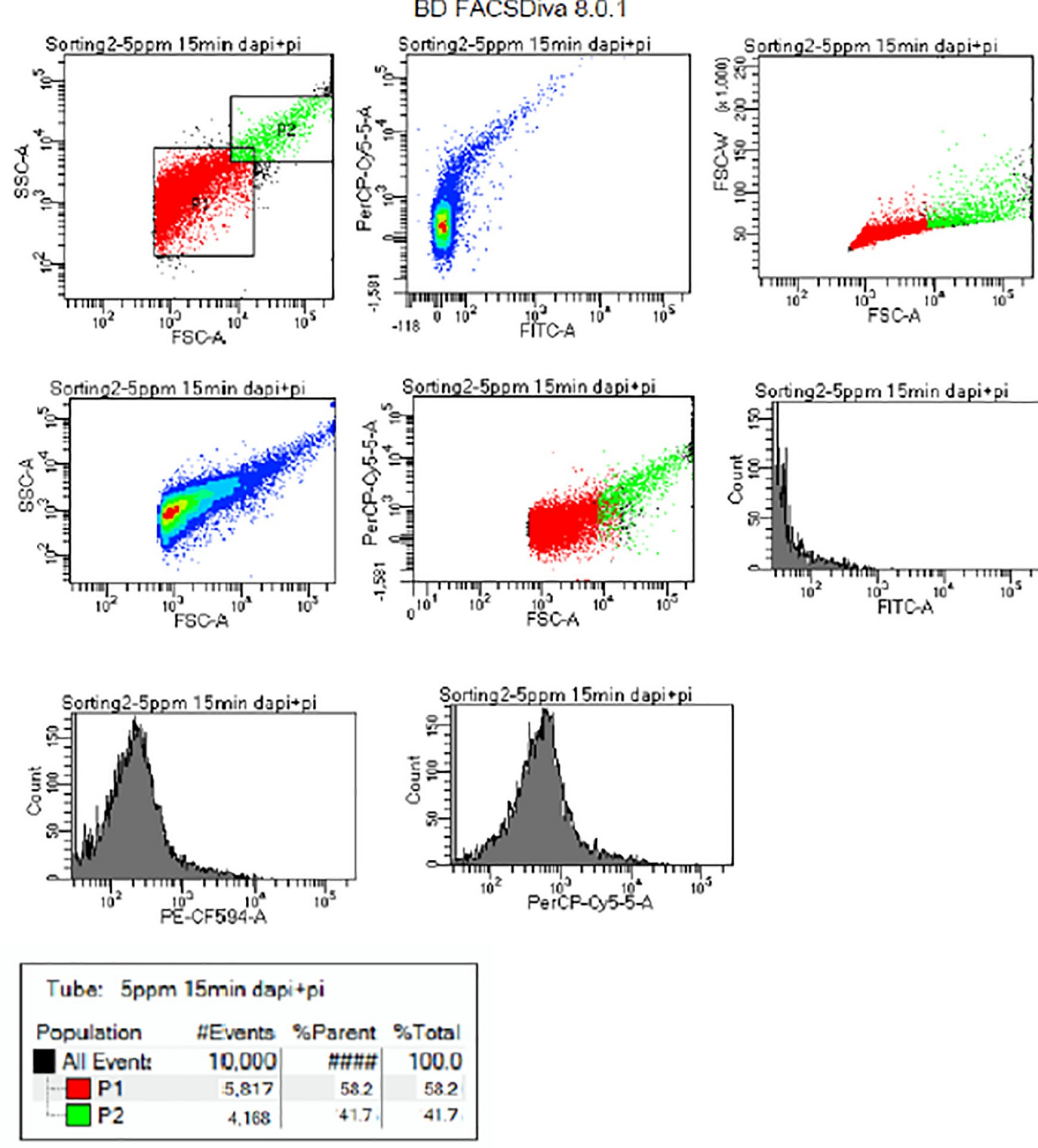
Flow cytometry histogram and dot plots showing viability of *Cryptosporidium* and *Giardia* at 15 seconds after exposure to 5 ppm of chlorine. Note: Samples of Region P1 and P2 represent gates for non-viable and viable (oo)cysts with P1 as side-scatter (SSC) against forward side-scatter (FSC).

Samples of flow cytometry histograms and dot plots showing viability of *Cryptosporidium* and *Giardia* are given in Figs 5 and 6. Fig 5 depicts exposure to UV irradiation after 20.8mJ/cm^2^ while Fig 6 presents exposure to 5 ppm of chlorine after 15 minutes. Other similar flow cytometry histograms and dot plots showing viability of *Cryptosporidium* and *Giardia* at different doses of UV and chlorine at different exposure times are available in the supporting document. In the supporting document, Figs A–E in S1 File present the diagrams for UV viability while Figs F to I in S1 File, Figs J–M in S1 File, and Figs N- Q in S1 File present the diagrams for 0.5 ppm, 2 ppm, and 5 ppm of chlorine respectively. Samples of Region P1 and P2 represent gates for non-viable and viable (oo)cysts with P1 as side-scatter (SSC) against forward side-scatter (FSC). Histograms represent percentages of non-viable (oo)cysts plotted with count against PE-CF594-A and PerCP0-Cy5-5-A. The gates P1 and P2 were used to illustrate a subgroup of data. Statistics was generated using the population and also to limit the number of events collected. The gated region shows the sample of mixed population of cells. The results of all the cytometry histogram and dot plots are presented in Table 6 for chlorine and Table 5 for UV.

### Comparison between Syto-9+PI and DAPI+PI vital dyes

Tables 3, 4, 5 and 6 present the comparison between the results of microscopy and flow cytometer. While the first 3 trials present the results using Syto-9+PI stains, the last 2 trials present results using DAPI+PI stains. The type of stain used for the experiment can have effect on the results as can be found in Fig 2. The figure presents the results of the two dyes differently. Syto-9+PI was used for trials 1 to 3 and DAPI+PI was used for trials 4 to 5. Syto-9+PI dyes gives slightly higher percentages than DAPI+PI. Therefore, Syto-9+PI is slightly more sensitive than DAPI+PI for *Cryptosporidium* and *Giardia*. This is notable because vital dyes may overestimate *Cryptosporidium* and *Giardia* in wastewater [48].

Largely, the results of mean percentage viability values using Syto-9+PI stains (trials 1 to 3) for (oo)cysts in Table 6 and Fig 4 were slightly higher than those using DAPI+PI stains (trials 4 to 5) at the initial stages of the experiment (Trials 1 to 3) and became comparable at Trials 4 and 5 because of overestimation of *Cryptosporidium* and *Giardia* as mentioned earlier [48]. The results of percentages of flow DAPI and PI of (oo)cysts were presented for different doses and exposure time for chlorine (Table 6). At 0.5 ppm, 85±3% were viable at 15 minutes and 17% were dead. This increased to 66±3% viable and 34% dead at the end of the trials at 120 minutes. The same results were recorded at 2 ppm. Nonetheless, at 5 ppm, 41±7% was viable and 59% dead at 15 minutes and 12±0% viable and 88% dead at the end of the trial (120 minutes). In Table 5, the efficacy of UV was amply demonstrated in the trials that only 3% were viable at 83.2 mJ/cm^2^ (160 minutes) and 97% dead. At 10.4 mJ/cm^2^(20 seconds), 11.0% were alive, which is lower than the highest dose of 5 ppm of chlorine (41%) at 15 minutes. Meanwhile after 20 seconds, there was a slight reduction in percentage alive with UV exposure unlike a significant decrement in percentage from 0 to 10seconds and 10 seconds to 20 seconds which was 56% and 88% respectively. Surprisingly, increase in the duration afterwards was not significant. [36] suggested oocysts has high resistance to UV irradiation and high UV dose may be required. Then again, UV may not remove all incidences of (oo)cysts as 3% was still alive after 83.2 mJ/cm^2^ (160 seconds).

## Conclusion and recommendations

This study presented the reactions of *Cryptosporidium* oocysts and *Giardia* cysts to UV irradiations and chlorine at different doses and exposure time. It has been found that effect of UV on *Giardia* cyst is more pronounced than the effect of chlorine at 0.5 ppm, 2 ppm and 5 ppm. More *Giardia* cysts became non-viable at lower chlorine concentration compared to *Cryptosporidium* oocysts. It takes a longer exposure time for *Cryptosporidium* oocysts reduction in viability. Increasing the chlorine doses resulted in the reduction of viability of *Cryptosporidium* and *Giardia*. This same trend was evident with UV irradiations. However, longer exposure time can also play significant role in the reduction in viability of *Cryptosporidium* and *Giardia* in wastewater treatment notably up to a threshold exposure time. At that threshold exposure time, further exposure has little or no effect on the viability. It was concluded in the study that *Cryptosporidium* oocyst is more resistant to both chlorine and UV irradiations than *Giardia* in wastewater treatment. The study suggests that *Cryptosporidium* may be resistant to chlorine treatment. Combined treatment of UV and chlorine may be needed to eradicate *Cryptosporidium* in water treatment in a shorter duration.

This study also investigated the use of two different vital dyes for determining the viability of *Cryptosporidium* and *Giardia* which are Syto-9+PI and DAPI+PI. It was found that Syto-9+PI gives higher mean percentage viability values than DAPI+PI in this study with wastewater. This shows that Syto-9+PI is more sensitive and may be better than DAPI+PI for estimating the presence of *Cryptosporidium* and *Giardia* in wastewater. The viability of *Cryptosporidium* and *Giardia* were determined after treatment through using microscopy and flow cytometer. It was found flow cytometer obtained higher percentage values than microscopy at higher doses for both chlorine and UV irradiations. Flow cytometer may be more sensitive than microscopy in detecting microorganisms especially *Cryptosporidium* and *Giardia* as flow cytometer has higher percentage values than microscopy in most of the results obtained in this study. It was further found that the treatment regime was more effective in the control where distilled water was used than in the wastewater. It was found that turbidity may affect water treatment using both chlorine and UV irradiation at different doses.

Further studies are suggested in evaluating the effect of water temperature on the UV irradiation for wastewater treatment. Furthermore, there is a need to perform this experiment on wastewater samples of different pH so as to establish the efficacy of UV irradiation and chlorine exposure at different pH levels. Different strains of *Cryptosporidium* and *Giardia* should be experimented on and compared. This will provide clarity on reactions of different strains of *Cryptosporidium* and *Giardia* to different doses and exposure time of chlorine and UV. More vital dyes should be evaluated to establish the accuracy of the results obtained in wastewater treatment using chlorine and UV.

## Supporting information

S1 File Flow cytometry histogram and dot plots showing viability of *Cryptosporidium* and *Giardia* at different durations after exposure to UV irradiation and different doses of chlorine. Fig A. Flow cytometry histogram and dot plots showing viability of *Cryptosporidium* and *Giardia* at 10 seconds after exposure to UV irradiation. Fig B. Flow cytometry histogram and dot plots showing viability of *Cryptosporidium* and *Giardia* at 20 seconds after exposure to UV irradiation. Fig C. Flow cytometry histogram and dot plots showing viability of *Cryptosporidium* and *Giardia* at 40 seconds after exposure to UV irradiation. Fig D. Flow cytometry histogram and dot plots showing viability of *Cryptosporidium* and *Giardia* at 80 seconds after exposure to UV irradiation. Fig E. Flow cytometry histogram and dot plots showing viability of *Cryptosporidium* and *Giardia* at 160 seconds after exposure to UV irradiation. Fig F. Flow cytometry histogram and dot plots showing viability of *Cryptosporidium* and *Giardia* at 15 minutes after exposure to 0.5 ppm of chlorine. Fig G. Flow cytometry histogram and dot plots showing viability of *Cryptosporidium* and *Giardia* at 30 minutes after exposure to 0.5 ppm of chlorine. Fig H. Flow cytometry histogram and dot plots showing viability of *Cryptosporidium* and *Giardia* at 60 minutes after exposure to 0.5 ppm of chlorine. Fig I. Flow cytometry histogram and dot plots showing viability of *Cryptosporidium* and *Giardia* at 120 minutes after exposure to 0.5 ppm of chlorine. Fig J. Flow cytometry histogram and dot plots showing viability of *Cryptosporidium* and *Giardia* at 15 minutes after exposure to 2 ppm of chlorine. Fig K. Flow cytometry histogram and dot plots showing viability of *Cryptosporidium* and *Giardia* at 30 minutes after exposure to 2 ppm of chlorine. Fig L. Flow cytometry histogram and dot plots showing viability of *Cryptosporidium* and *Giardia* at 60 minutes after exposure to 2 ppm of chlorine. Fig M. Flow cytometry histogram and dot plots showing viability of *Cryptosporidium* and *Giardia* at 120 minutes after exposure to 2 ppm of chlorine. Fig N. Flow cytometry histogram and dot plots showing viability of *Cryptosporidium* and *Giardia* at 15 minutes after exposure to 5 ppm of chlorine. Fig O. Flow cytometry histogram and dot plots showing viability of *Cryptosporidium* and *Giardia* at 30 minutes after exposure to 5 ppm of chlorine. Fig P. Flow cytometry histogram and dot plots showing viability of *Cryptosporidium* and *Giardia* at 60 minutes after exposure to 5 ppm of chlorine. Fig Q. Flow cytometry histogram and dot plots showing viability of *Cryptosporidium* and *Giardia* at 120 minutes after exposure to 5 ppm of chlorine.
(PDF)
