# Supplementary material for: Efficiency of chlorine and UV in the inactivation of Cryptosporidium and Giardia in wastewater
Source: PLoS One. 2019 May 13;14(5):e0216040. doi: 10.1371/journal.pone.0216040 (PMC6513095; doi:10.1371/journal.pone.0216040)
Supplement: S1 File — Fig A. Flow cytometry histogram and dot plots showing viability of Cryptosporidium and Giardia at 10 seconds after exposure to UV irradiation. Fig B. Flow cytometry histogram and dot plots showing viability of Cryptosporidium and Giardia at 20 seconds after exposure to UV irradiation. Fig C. Flow cytometry histogram and dot plots showing viability of Cryptosporidium and Giardia at 40 seconds after exposure to UV irradiation. Fig D. Flow cytometry histogram and dot plots showing viability of Cryptosporidium and Giardia at 80 seconds after exposure to UV irradiation. Fig E. Flow cytometry histogram and dot plots showing viability of Cryptosporidium and Giardia at 160 seconds after exposure to UV irradiation. Fig F. Flow cytometry histogram and dot plots showing viability of Cryptosporidium and Giardia at 15 minutes after exposure to 0.5 ppm of chlorine. Fig G. Flow cytometry histogram and dot plots showing viability of Cryptosporidium and Giardia at 30 minutes after exposure to 0.5 ppm of chlorine. Fig H. Flow cytometry histogram and dot plots showing viability of Cryptosporidium and Giardia at 60 minutes after exposure to 0.5 ppm of chlorine. Fig I. Flow cytometry histogram and dot plots showing viability of Cryptosporidium and Giardia at 120 minutes after exposure to 0.5 ppm of chlorine. Fig J. Flow cytometry histogram and dot plots showing viability of Cryptosporidium and Giardia at 15 minutes after exposure to 2 ppm of chlorine. Fig K. Flow cytometry histogram and dot plots showing viability of Cryptosporidium and Giardia at 30 minutes after exposure to 2 ppm of chlorine. Fig L. Flow cytometry histogram and dot plots showing viability of Cryptosporidium and Giardia at 60 minutes after exposure to 2 ppm of chlorine. Fig M. Flow cytometry histogram and dot plots showing viability of Cryptosporidium and Giardia at 120 minutes after exposure to 2 ppm of chlorine. Fig N. Flow cytometry histogram and dot plots showing viability of Cryptosporidium and Giardia at 15 minut [file pone.0216040.s001.pdf]

BD FACSDiva 8.0.1

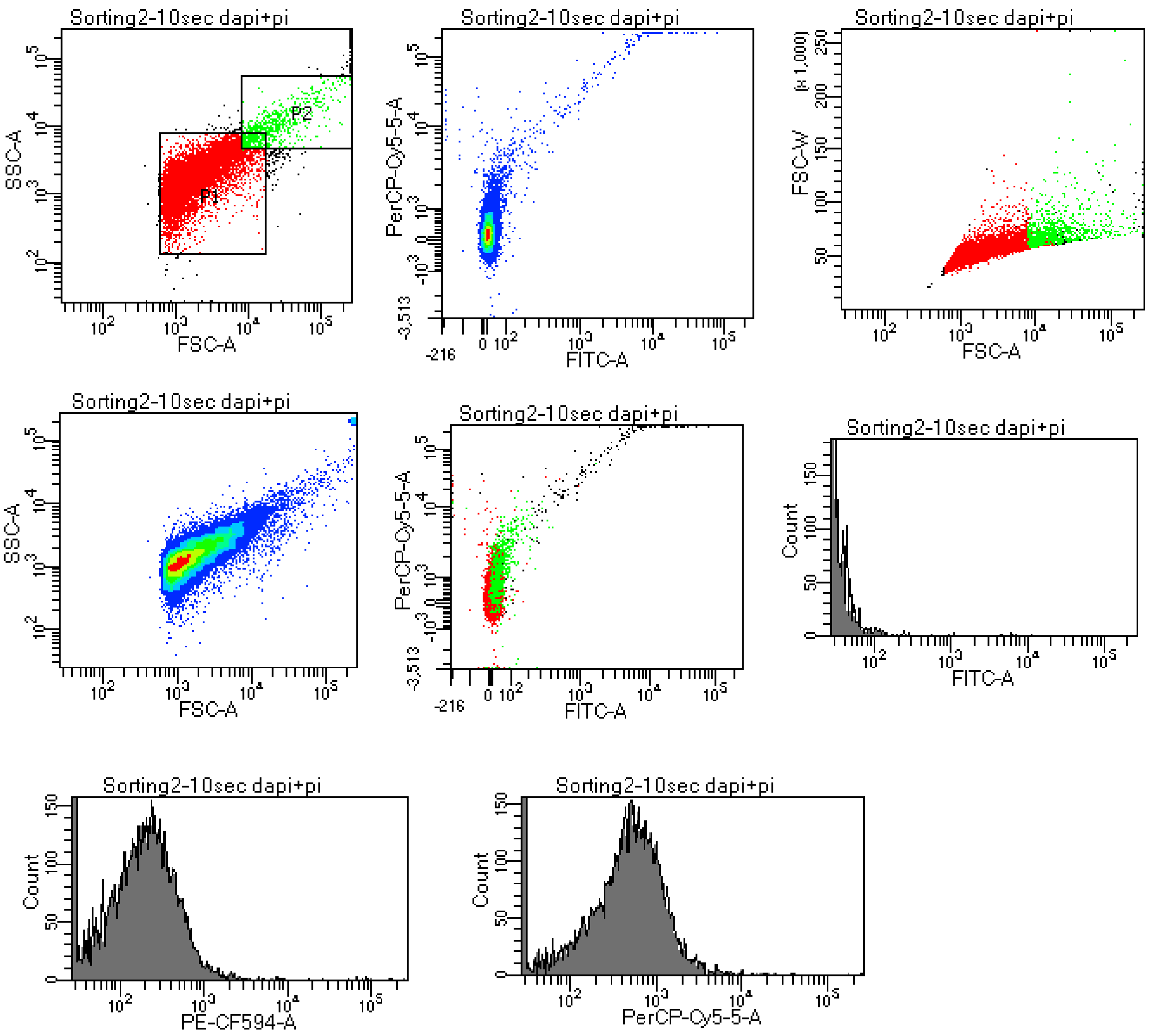

Tube: 10sec dapi+pi

Population	#Events	%Parent	%Total
All Events	10,000	####	100.0
P1	5,661	56.6	56.6
P2	4,324	43.2	43.2

BD FACSDiva 8.0.1

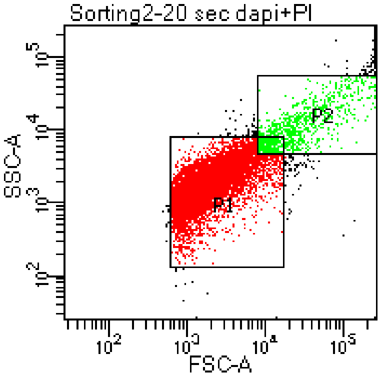

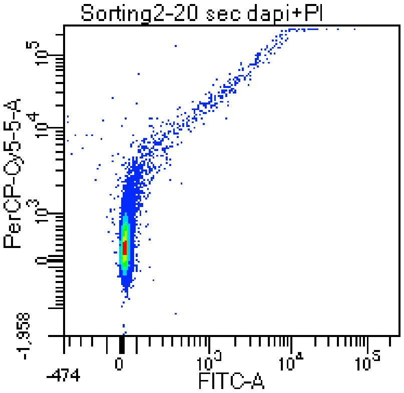

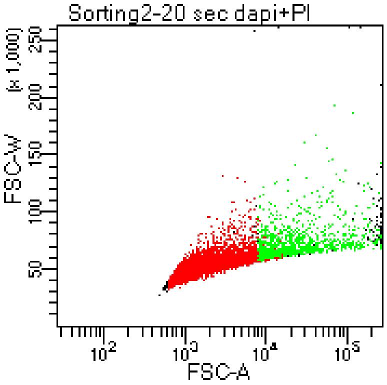

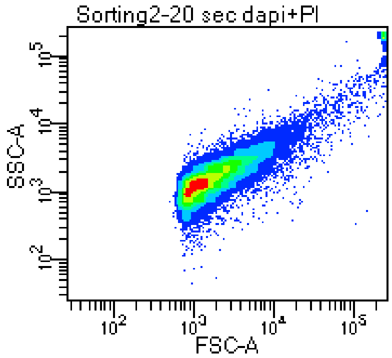

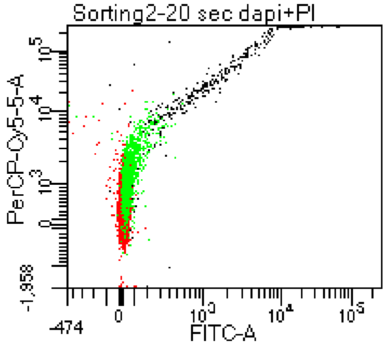

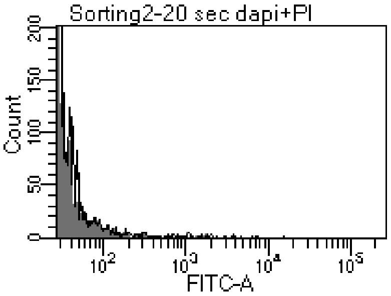

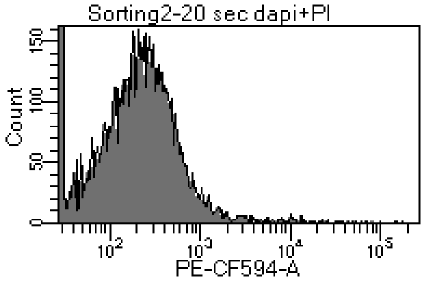

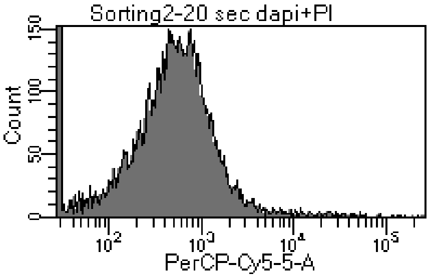

Tube: 20 sec dapi+PI

Population	#Events	%Parent	%Total
All Events	10,000	####	100.0
P1	8,827	88.3	88.3
P2	1,102	11.0	11.0

BD FACSDiva 8.0.1

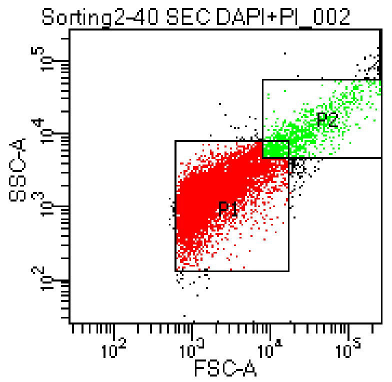

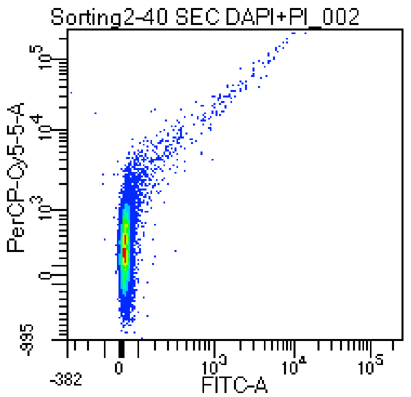

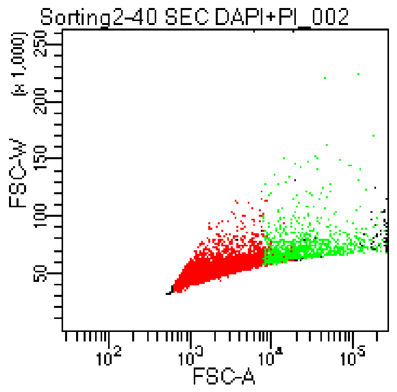

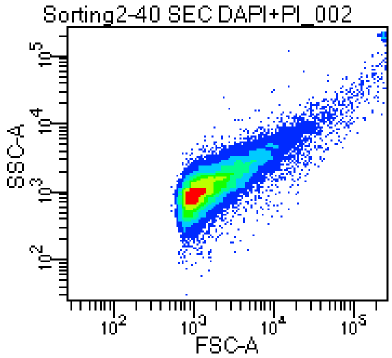

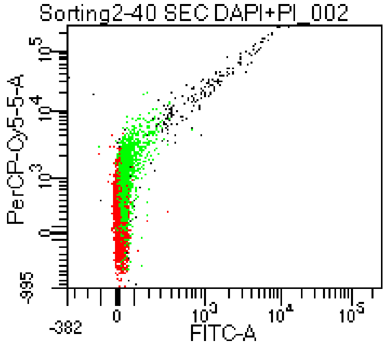

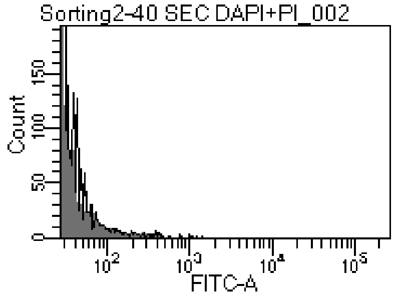

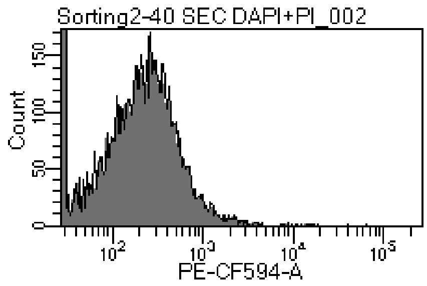

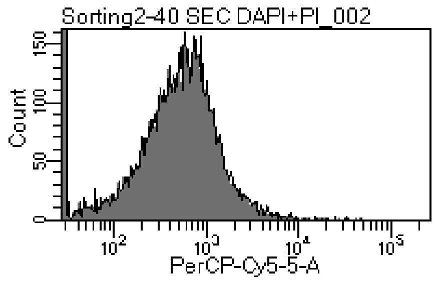

Tube: 40 SEC DAPI+PI_002

Population	#Events	%Parent	%Total
All Events	10,000	####	100.0
P1	8,997	90.0	90.0
P2	985	9.8	9.8

BD FACSDiva 8.0.1

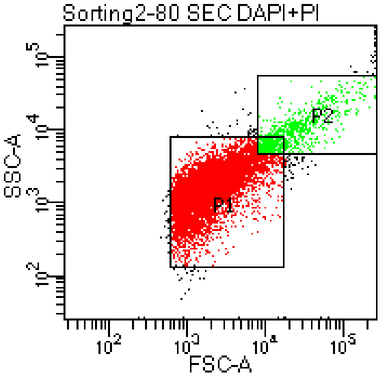

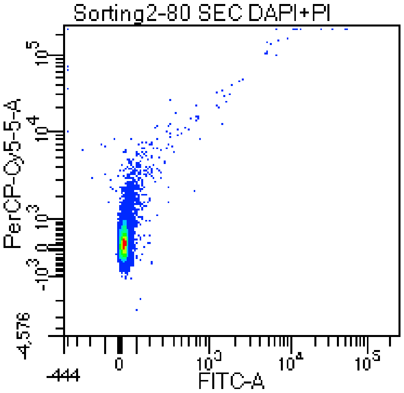

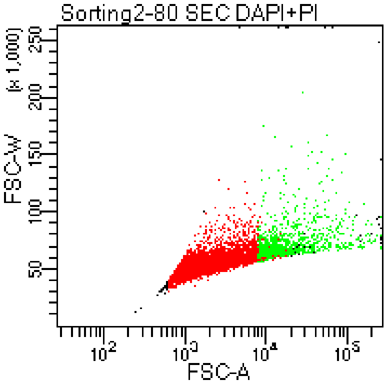

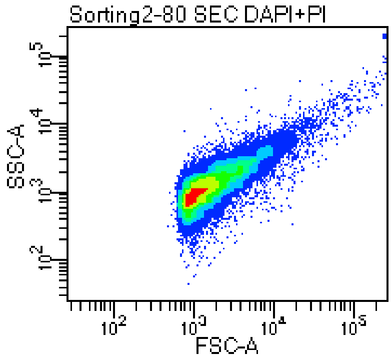

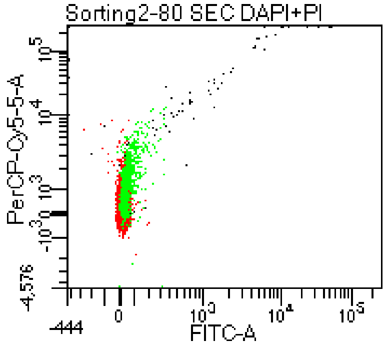

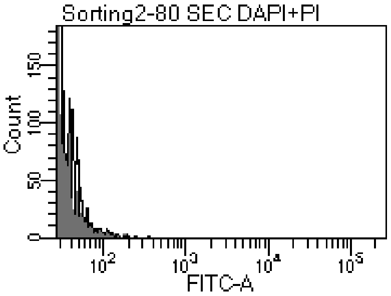

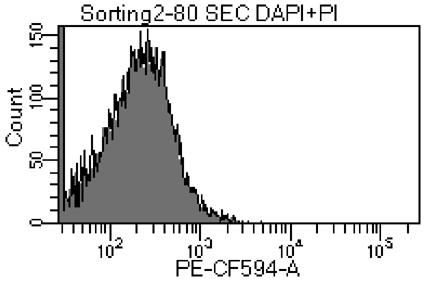

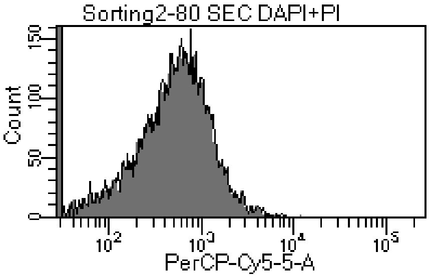

Tube: 80 SEC DAPI+PI

Population	#Events	%Parent	%Total
All Events	10,000	####	100.0
P1	9,323	93.2	93.2
P2	854	8.5	8.5

BD FACSDiva 8.0.1

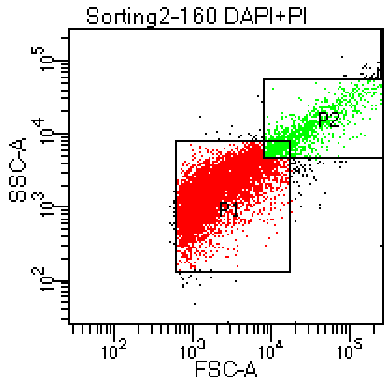

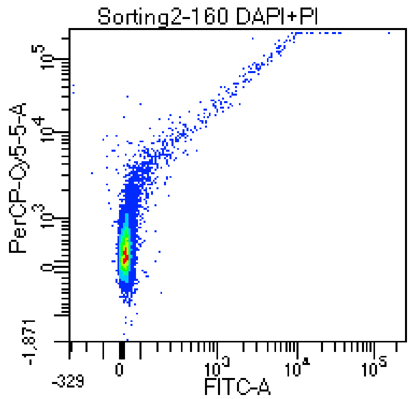

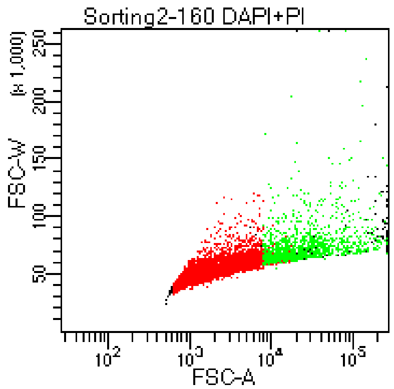

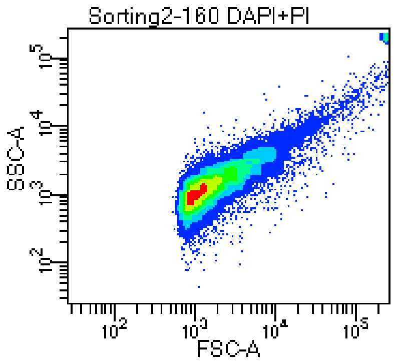

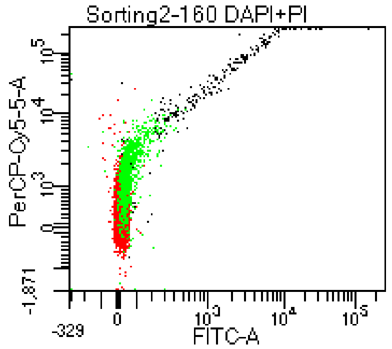

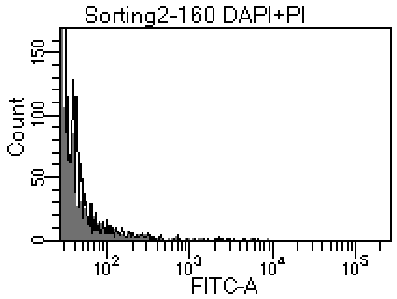

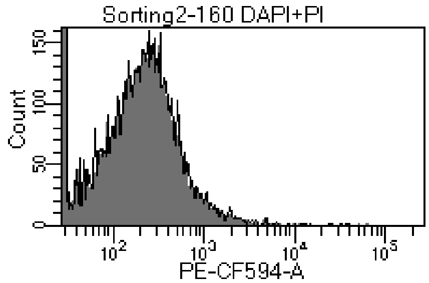

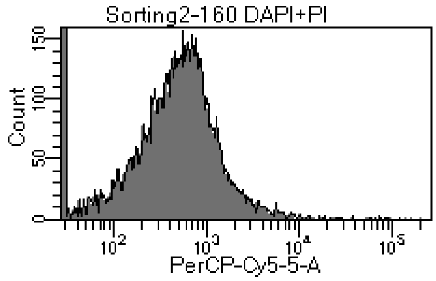

Tube: 160 DAPI+PI

Population	#Events	%Parent	%Total
All Events	10,000	####	100.0
P1	9,783	97.8	97.8
P2	3,114	3.1	3.1

BD FACSDiva 8.0.1

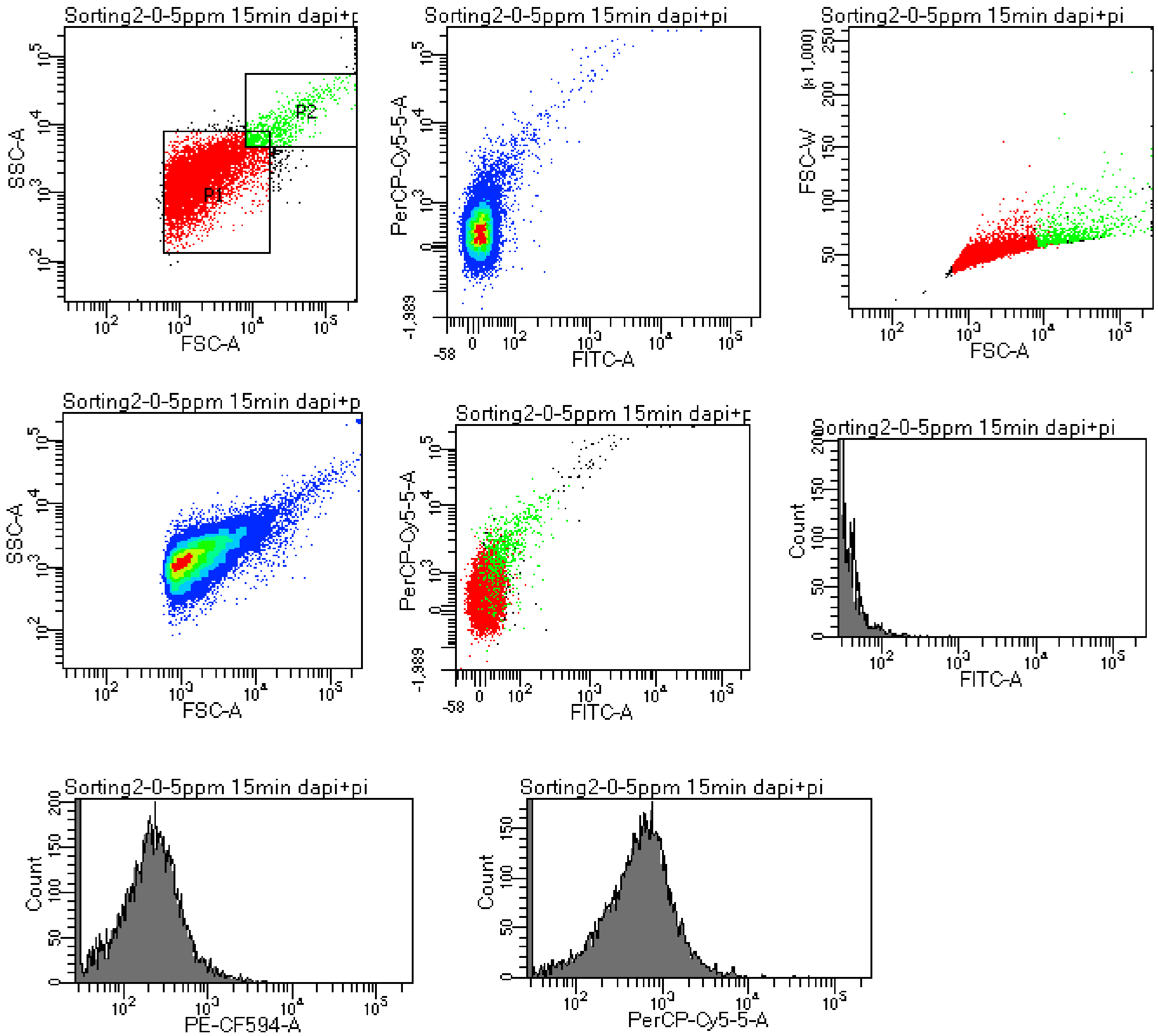

Tube: 0-5ppm 15min dapi+pi

Population	#Events	%Parent	%Total
All Events	10,000	####	100.0
P1	1,612	16.1	16.1
P2	8323	83.2	83.2

Printed on: Fri Aug 18, 2017 03:27:17 CAT

BD FACSDiva 8.0.1

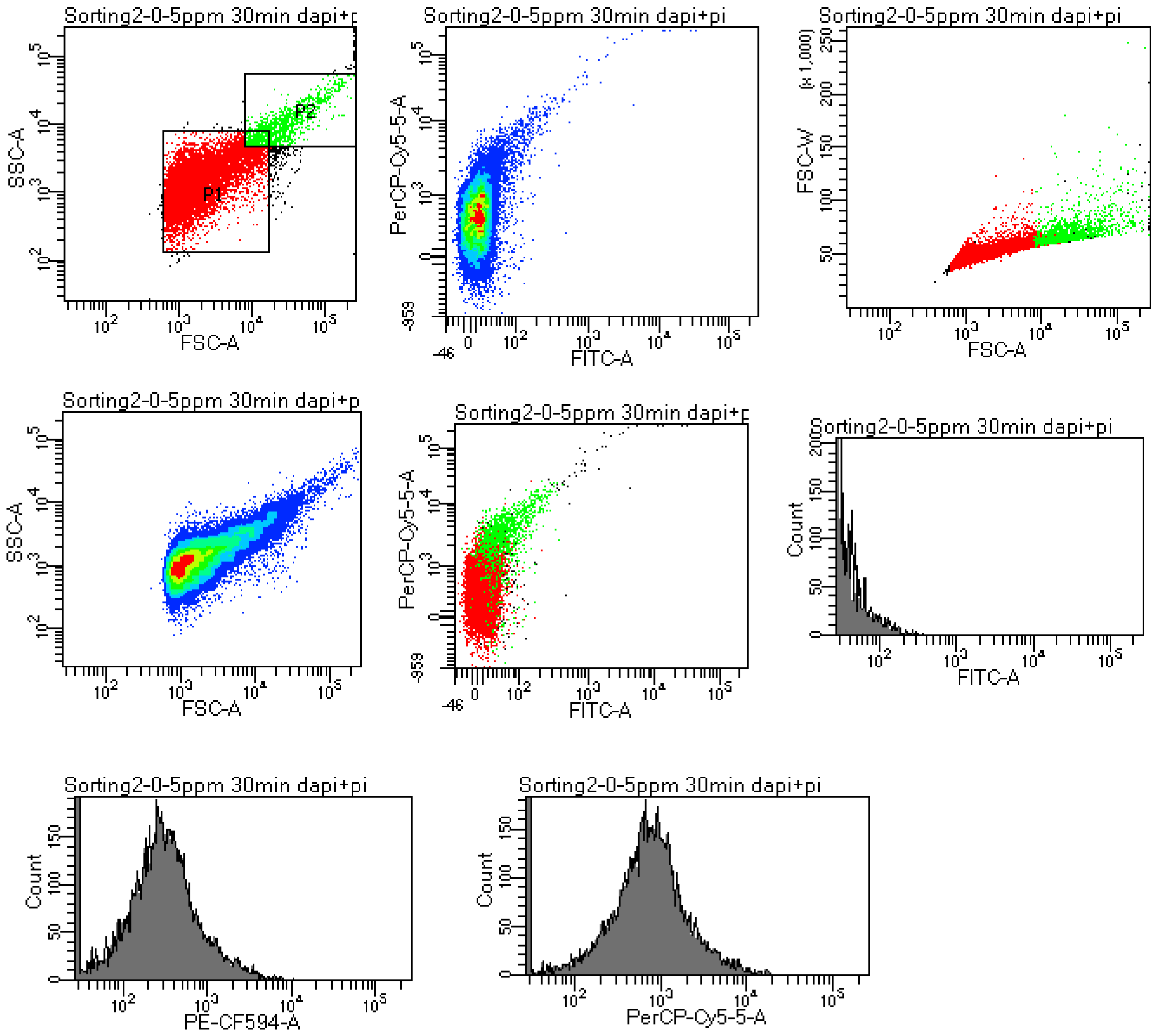

Tube: 0-5ppm 30min dapi+pi

Population	#Events	%Parent	%Total
All Events	10,000	####	100.0
P1	2,211	22.1	22.1
P2	7,991	79.9	79.9

BD FACSDiva 8.0.1

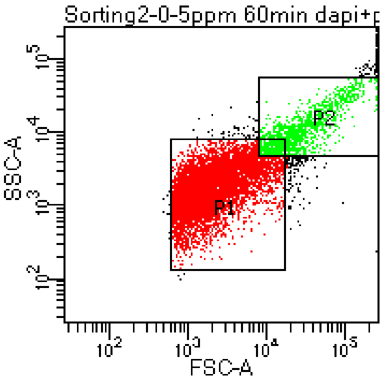

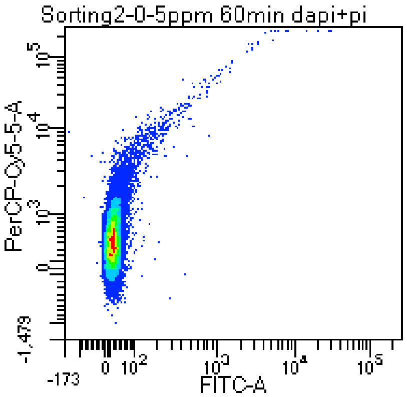

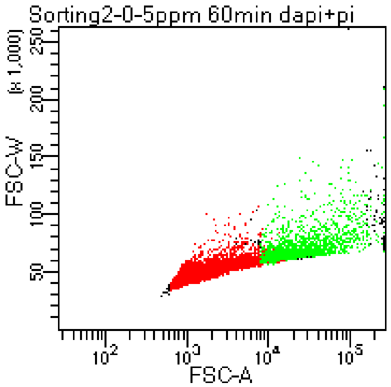

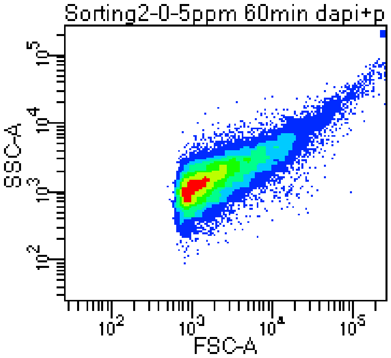

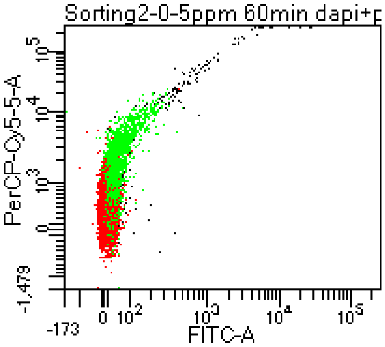

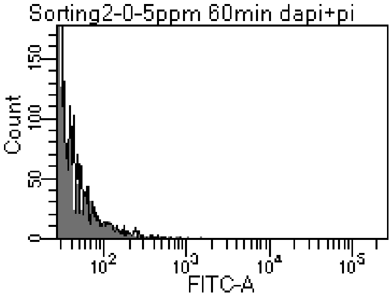

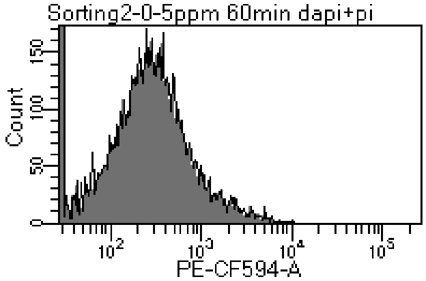

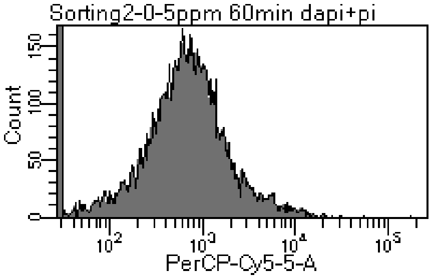

Tube: 0-5ppm 60min dapi+pi

Population	#Events	%Parent	%Total
All Events	10,000	####	100.0
P1	3,011	30.1	30.1
P2	7,164	71.6	71.6

BD FACSDiva 8.0.1

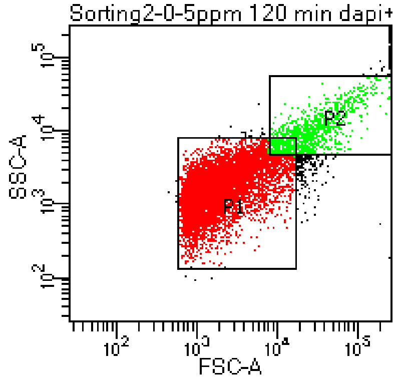

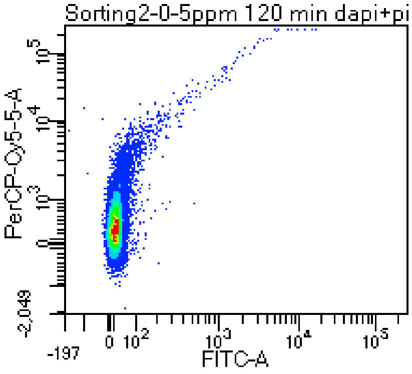

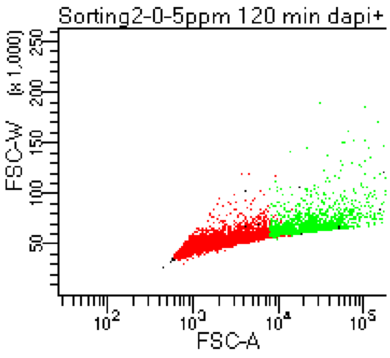

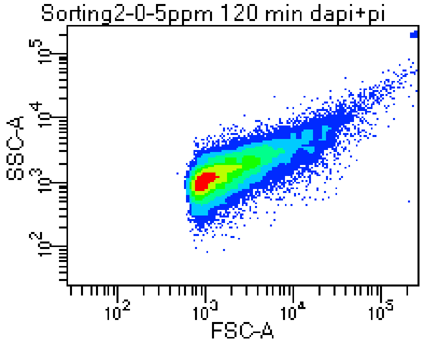

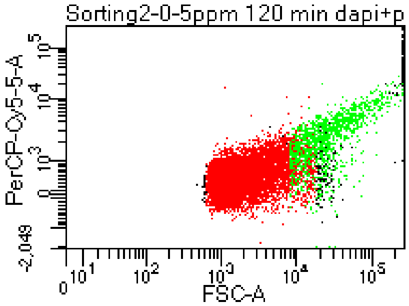

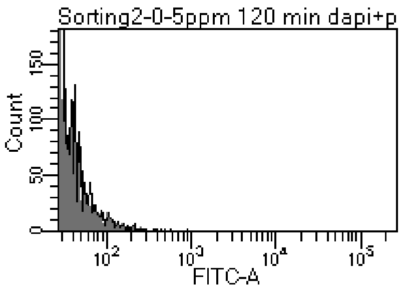

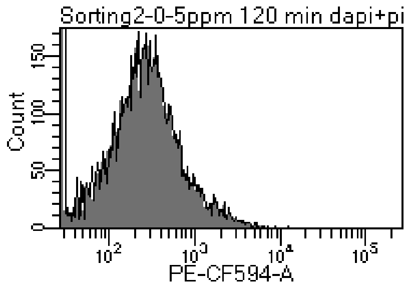

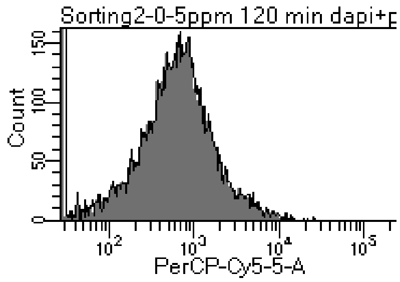

Tube: 0-5ppm 120 min dapi+pi

Population	#Events	%Parent	%Total
All Events	10,000	####	100.0
P1	3,372	33.7	33.7
P2	6,713	67.1	67.1

BD FACSDiva 8.0.1

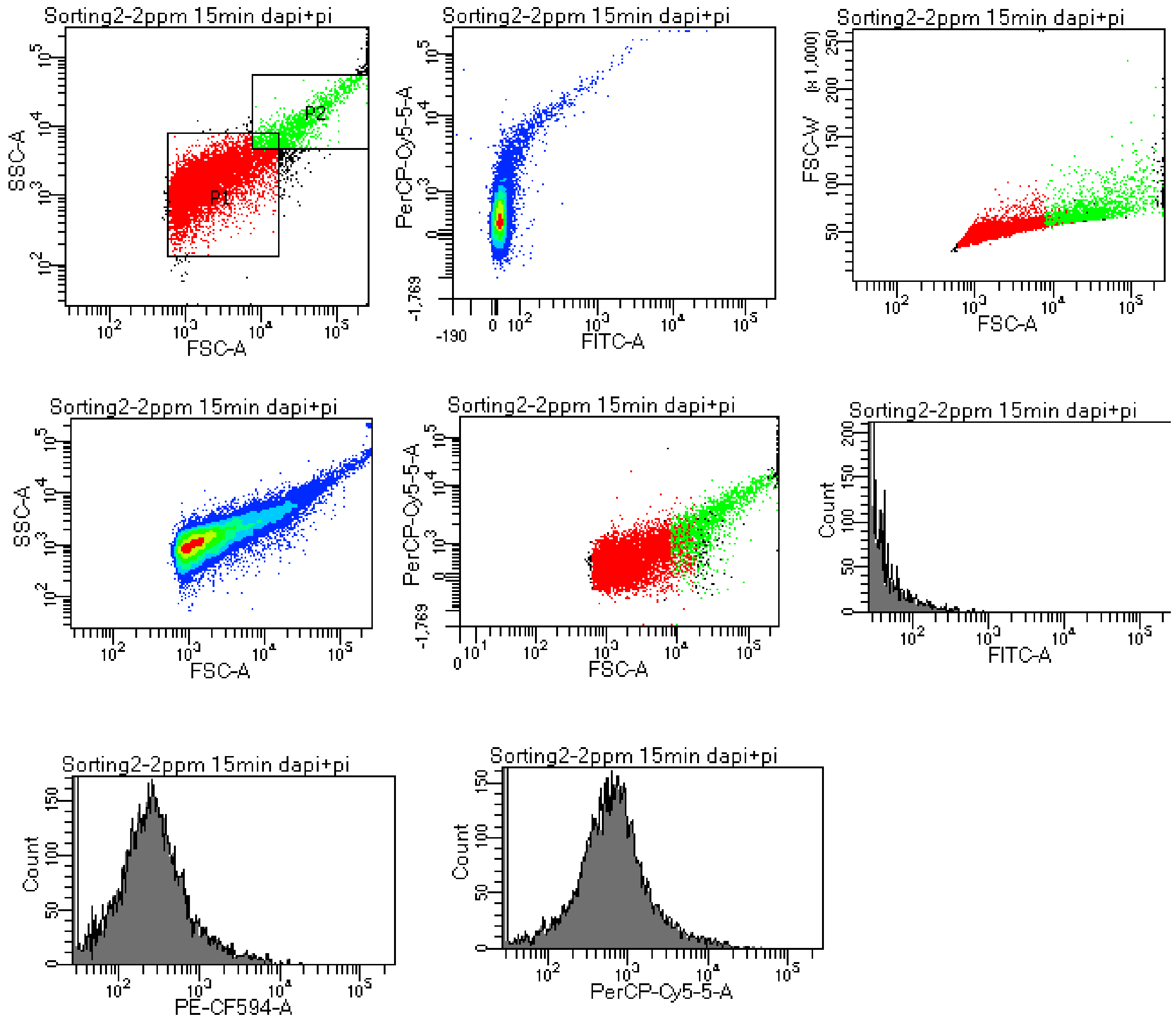

Tube: 2ppm 15min dapi+pi

Population	#Events	%Parent	%Total
All Events	10,000	####	100.0
P1	1,387	13.9	13.9
P2	8,621	86.2	86.2

BD FACSDiva 8.0.1

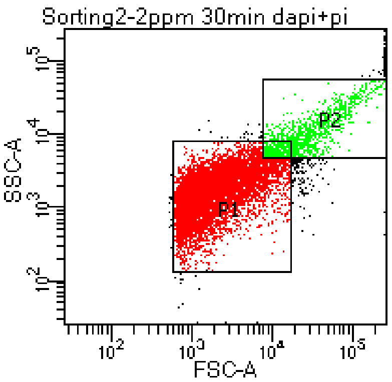

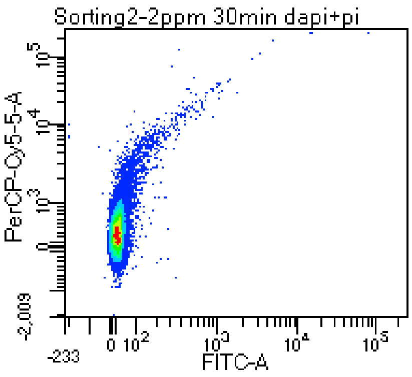

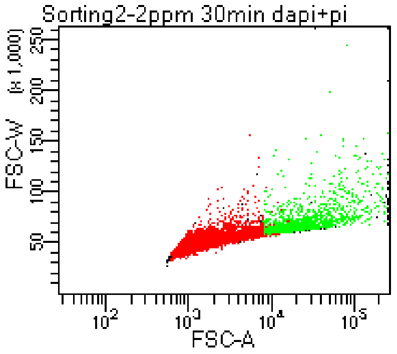

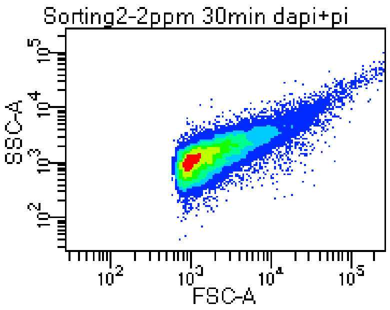

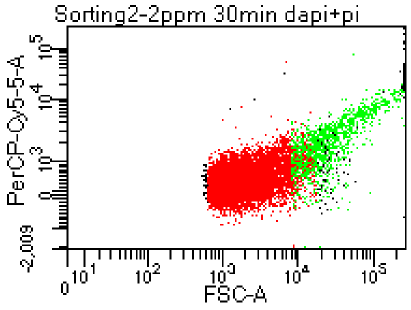

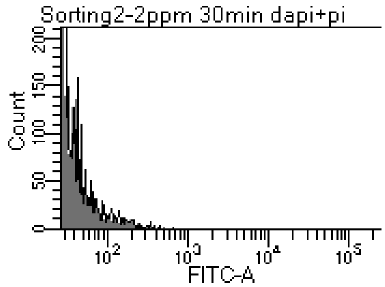

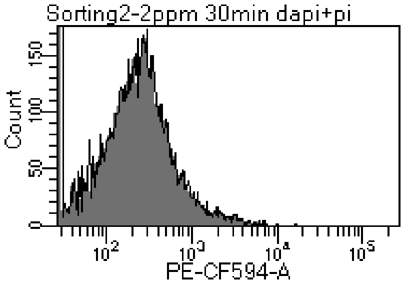

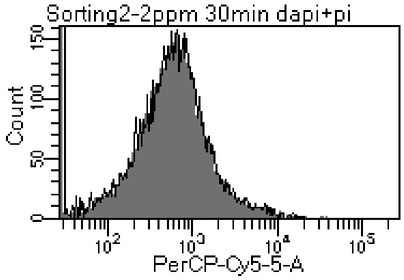

Tube: 2ppm 30min dapi+pi

Population	#Events	%Parent	%Total
All Events	10,000	####	100.0
P1	2,922	29.2	29.2
P2	7,163	71.6	71.6

BD FACSDiva 8.0.1

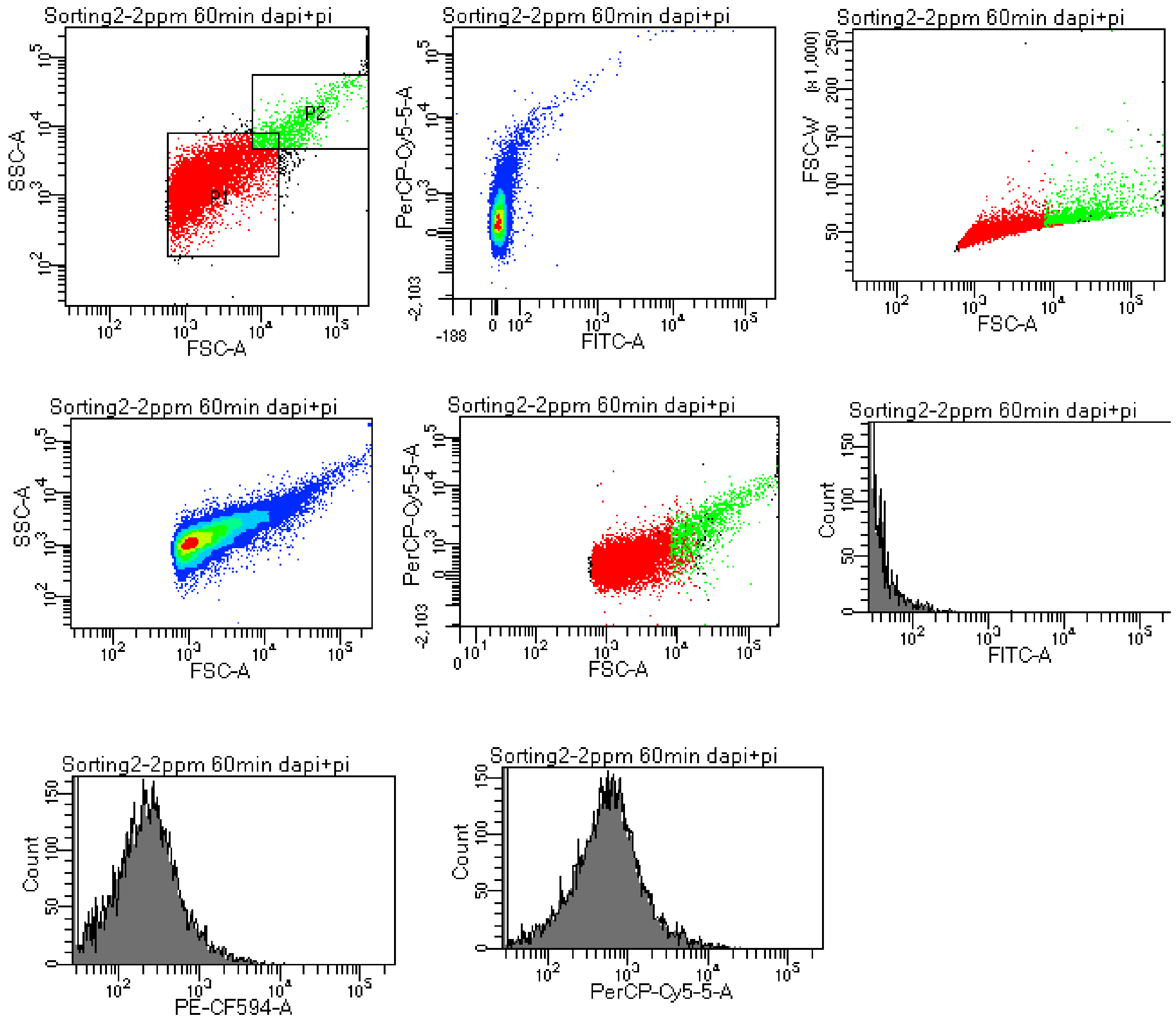

Tube: 2ppm 60min dapi+pi			
Population	#Events	%Parent	%Total
All Events	10,000	####	100.0
P1	3,331	33.3	33.3
P2	6,770	67.7	67.7

BD FACSDiva 8.0.1

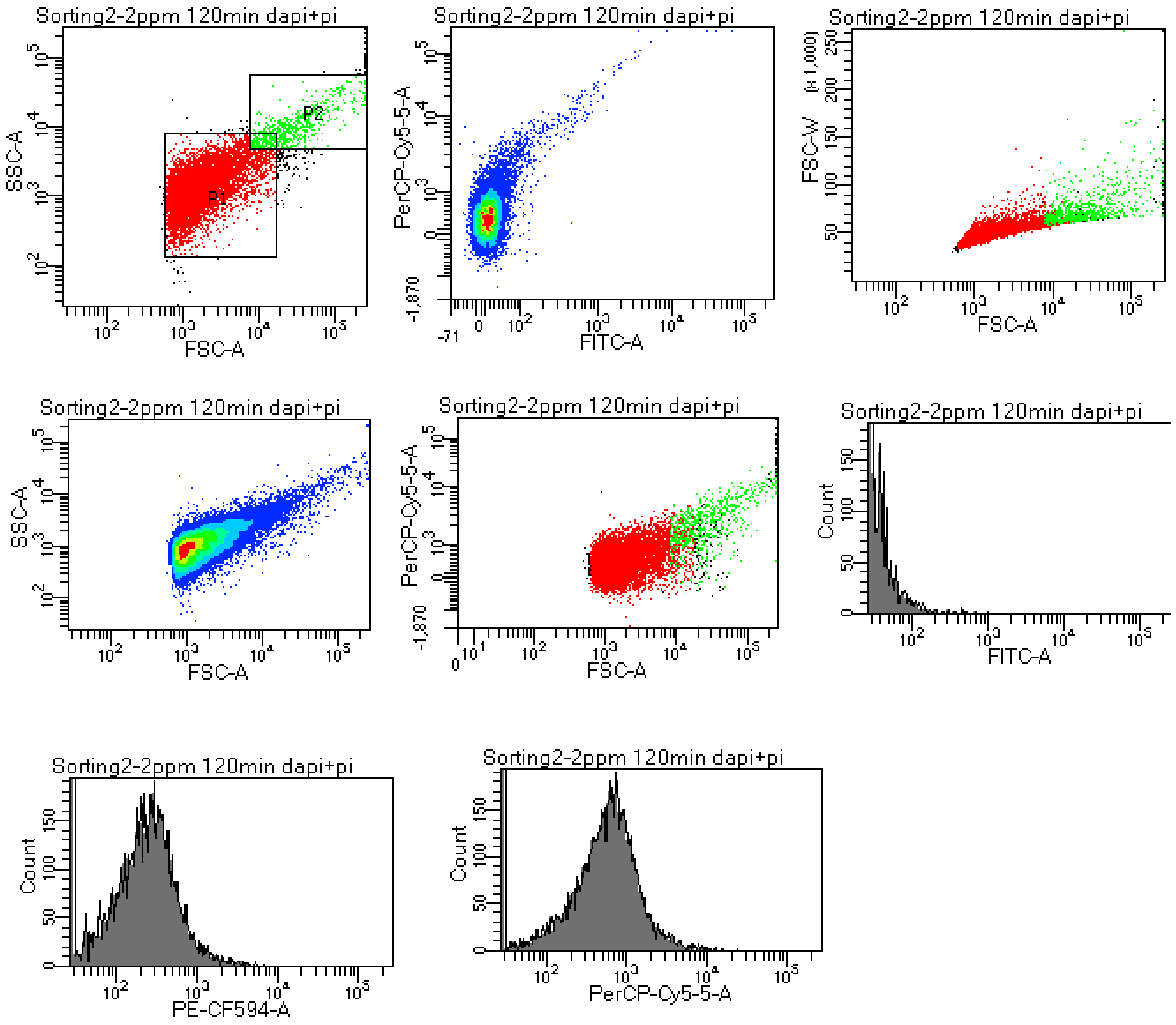

Tube: 2ppm 120min dapi+pi			
Population	#Events	%Parent	%Total
All Events	10,000	####	100.0
P1	4,241	42.4	42.4
P2	5,754	57.5	57.5

BD FACSDiva 8.0.1

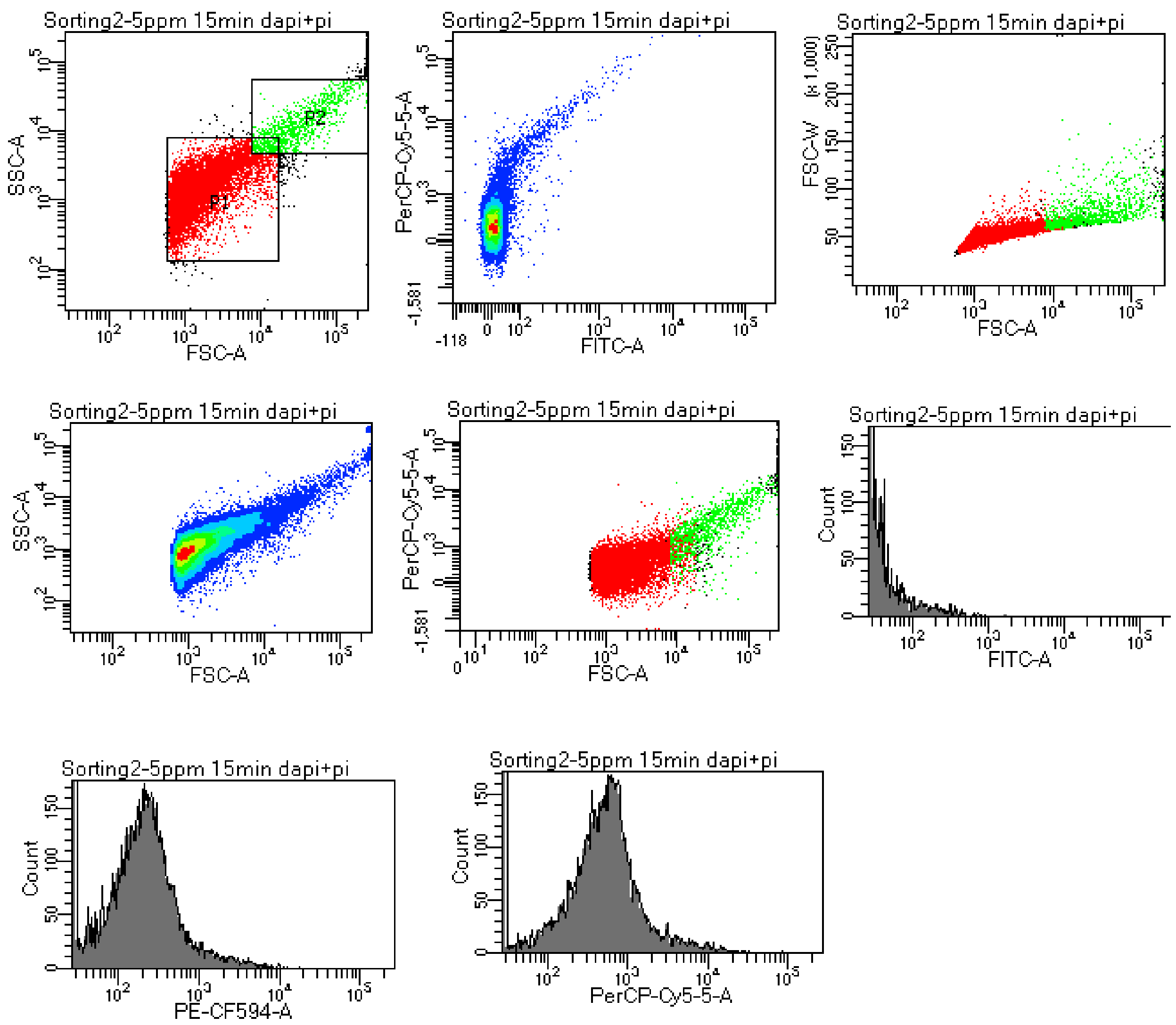

Tube: 5ppm 15min dapi+pi

Population	#Events	%Parent	%Total
All Events	10,000	####	100.0
P1	5,817	58.2	58.2
P2	4,168	41.7	41.7

BD FACSDiva 8.0.1

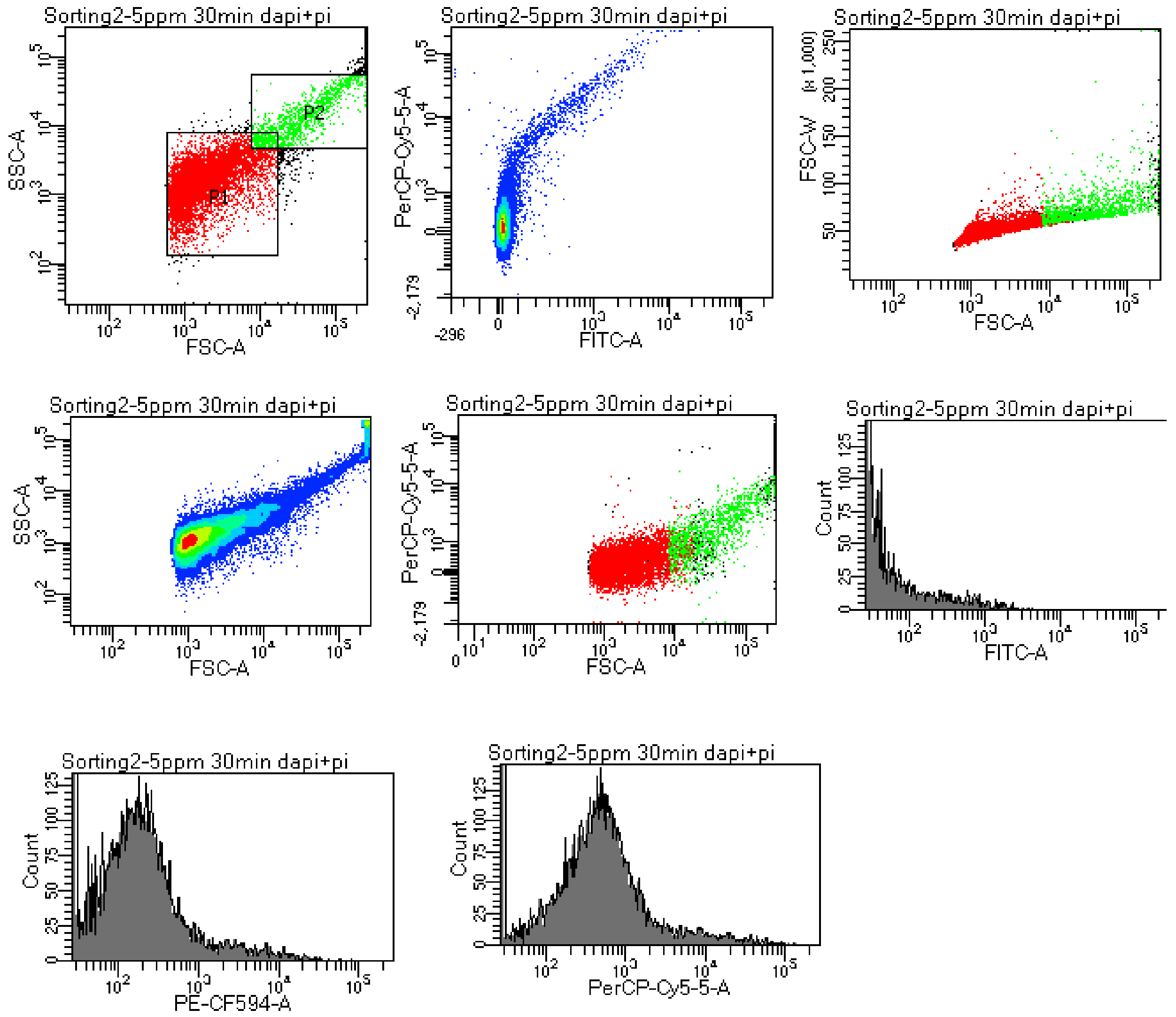

Tube: 5ppm 30min dapi+pi

Population	#Events	%Parent	%Total
All Events	10,000	####	100.0
P1	6,327	63.3	63.3
P2	3,298	33.0	33.0

BD FACSDiva 8.0.1

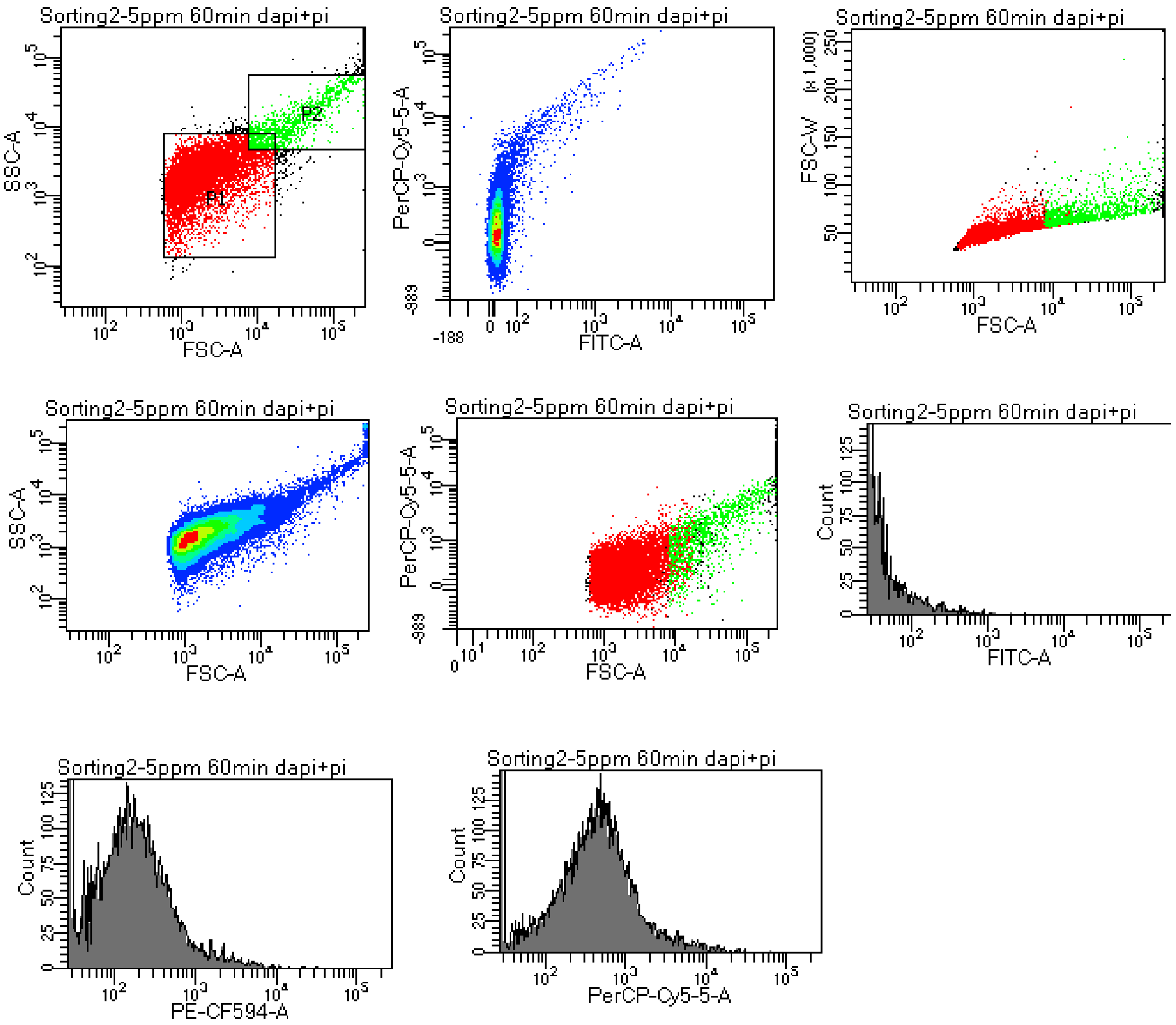

Tube: 5ppm 60min dapi+pi

Population	#Events	%Parent	%Total
All Events	10,000	####	100.0
P1	7,771	77.7	77.7
P2	2,127	21.3	21.3

BD FACSDiva 8.0.1

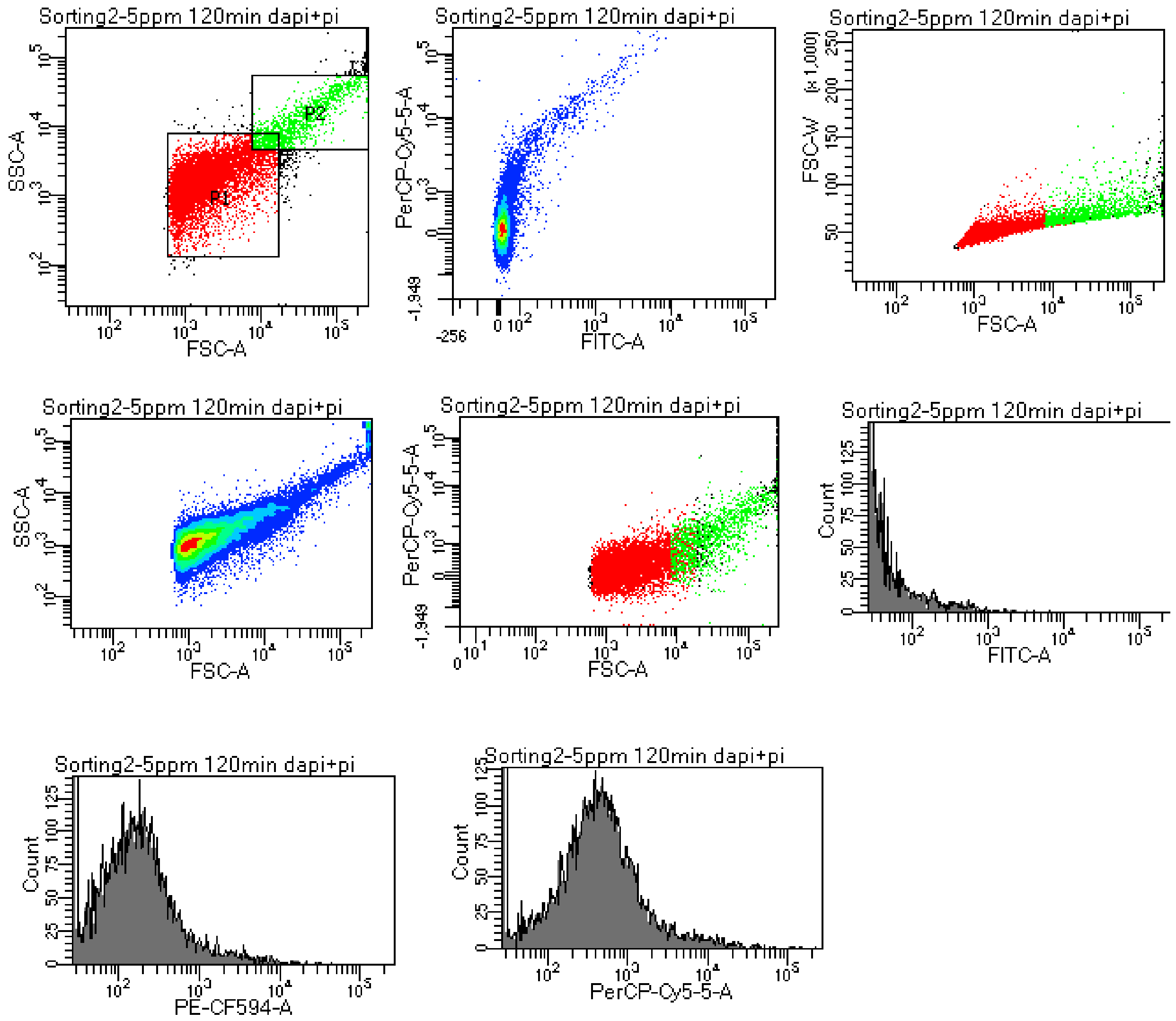

Tube: 5ppm 120min dapi+pi

Population	#Events	%Parent	%Total
All Events	10,000	####	100.0
P1	8,553	85.5	85.5
P2	1,265	12.6	12.6
